# Feasibility of self-reported health related quality of life assessment with older people in residential care: insights from the application of eye tracking technology

**DOI:** 10.1007/s11136-023-03488-w

**Published:** 2023-07-20

**Authors:** Rachel Milte, Matthew Crocker, Kiri Lay, Julie Ratcliffe, Brendan Mulhern, Richard Norman, Rosalie Viney, Jyoti Khadka

**Affiliations:** 1 Caring Futures Institute, Flinders University, GPO Box 2100, Adelaide, SA 5001 Australia; 2 Centre for Health Economics Research and Evaluation, University of Technology Sydney, Ultimo, NSW Australia; 3 Curtin School of Population Health, Curtin University, Bentley, WA Australia

**Keywords:** Quality of life, EQ-5D-5L, Cognitive impairment, Older adult, Eye-tracking, Feasibility

## Abstract

**Purpose:**

Increasingly there are calls to routinely assess the health-related quality of life (HRQoL) of older people receiving aged care services, however the high prevalence of dementia and cognitive impairment remains a challenge to implementation. Eye-tracking technology facilitates detailed assessment of engagement and comprehension of visual stimuli, and may be useful in flagging individuals and populations who cannot reliably self-complete HRQoL instruments. The aim of this study was to apply eye-tracking technology to provide insights into self-reporting of HRQoL among older people in residential care with and without cognitive impairment.

**Methods:**

Residents (*n* = 41), recruited based on one of three cognition subgroups (no, mild, or moderate cognitive impairment), completed the EQ-5D-5L on a computer with eye tracking technology embedded. Number and length of fixations (i.e., eye gaze in seconds) for key components of the EQ-5D-5L descriptive system were calculated.

**Results:**

For all dimensions, participants with no cognitive impairment fixated for longer on the Area of Interest (AOI) for the response option they finally chose, relative to those with mild or moderate cognitive impairment. Participants with cognitive impairment followed similar fixation patterns to those without. There was some evidence that participants with cognitive impairment took longer to complete and spent relatively less time attending to the relevant AOIs, but these differences did not reach statistical significance generally.

**Conclusions:**

This exploratory study applying eye tracking technology provides novel insights and evidence of the feasibility of self-reported HRQoL assessments in older people in aged care settings where cognitive impairment and dementia are highly prevalent.

**Supplementary Information:**

The online version contains supplementary material available at 10.1007/s11136-023-03488-w.

## Plain English summary

The world’s population is ageing and increasingly there is pressure on systems designed to care and support older people in many countries. There are calls to improve aged care systems and protect vulnerable older people from poor quality care. Asking older people to complete questionnaires about their quality of life could provide information about high and low quality residential aged care services as part of quality assessment programs. However, completing these questionnaires with residents with cognitive impairment or dementia is potentially challenging. This study uses eye-tracking technology with residents while they complete a quality of life questionnaire. We use eye-tracking technology to understand more about how residents go about filling out these questionnaires, and any differences between people with and without cognitive impairment when they fill out these questionnaires. We found that while those with cognitive impairment generally approached the questionnaires in a similar way, they took longer to complete the questionnaires and were distracted away from the wording parts of the questionnaire more often. These results could be used to help improve the layout and content of questionnaires in the future for older people in residential care, and to identify older people who can and can not reliably answer these questionnaires

## Background

Globally over the next 30 years the number of older people (aged 65 years and above) is expected to more than double [1]. Over 200 million older people currently reside in Europe and North America, and the proportion of older people in developing nations is expected to increase exponentially over the coming decades. This demographic transformation poses major future challenges for the financing and organisation of aged care and health systems across many countries [2–6]. Consequently, there are increasing calls for improvements in quality assessment processes to protect quality of life (QoL) and wellbeing among older people accessing aged care. On-going monitoring of quality of care indicators and public reporting of QoL from the perspective of the older people receiving aged care services is a key component of this.

More than 50% of current residents in Australian residential aged care facilities have a dementia diagnosis [7]. The increasing prevalence of cognitive impairment and dementia poses a significant challenge for routine assessment and reporting of QoL. Where older people have severe cognitive impairment and dementia, reliable self-reporting of QoL using validated instruments is unlikely to be possible [8, 9]. In cases of mild to moderate cognitive impairment and dementia, there is ongoing debate surrounding the ability to reliably self-report QoL [10, 11]. Despite increasing calls for inclusivity, in the absence of definitive guidance on this topic, proxy assessment of QoL by family members or care staff is often used for older people in aged care settings, regardless of the level of cognitive impairment [12–14].

Decisions relating to self or proxy completion may potentially significantly impact the results from QoL assessments. Empirical comparison studies, incorporating both self- and proxy-assessed QoL for older people, have generally found poor to moderate levels of agreement, with proxies tending to report lower scores for the person than the person themselves [8, 15]. This knowledge, along with increasing recognition of the agency of older people has led to agreement of the importance of striving for self-assessment of QoL by the older person themselves wherever possible [14, 16]. However, currently, the decision on whether to seek self-or proxy-report of QoL for older people with varying levels of cognitive impairment tends to be made by researchers/clinical teams, with little guidance (either from instrument developers or in the peer-reviewed literature) regarding the optimal approach to adopt.

The EQ-5D is the world’s most widely applied generic preference-based measure of health related quality of life (HRQoL) [17]. To date it has predominantly been used with adults in health system settings for measuring and valuing HRQoL as a component of health technology assessment, economic evaluation and quality assessment [18, 19]. Some further background information on the EQ-5D can be found in the Supplementary Information files. Over the past decade, there has increasingly been interest in using the EQ-5D instrument with people living with cognitive impairment and dementia. The EQ-5D offers some potential advantages as a measure, including its wide-spread use allowing comparison of the impact of dementia and potential interventions with other acute or chronic conditions. However, the feasibility, acceptability and psychometric properties of the EQ-5D among people living with cognitive impairment and dementia is debated. Some studies have found good acceptability and reliability of the EQ-5D-3L among people with mild to moderate dementia, but poorer self-completion of the instrument among people with moderate or severe dementia [20–22]. Engel et al. [16] evaluated the face and content validity among people living with dementia, and identified that while feasible there were a number of potential challenges in interpretation for people living with dementia, for example the double-barrelled questions for the domains Anxiety/Depression and Pain/Discomfort. A recent review identified the EQ-5D as the most commonly used instrument for measuring outcomes in economic evaluations in aged care settings [18].

More generally, a small but growing literature has shown potential errors in responses to self-report questionnaires (such as HRQoL instruments) is associated with cognitive impairment. For example, there is evidence that response patterns such as skipped questions, responses which are less internally consistent, higher levels of acquiescent responses (where participants accept the default statement without re-evaluating their current status against the default) and Guttman errors (where participants agree to a strongly worded item and then do not to the same level or higher to a similar but more moderately worded item) are significantly associated with risk of decline in cognitive function, developing dementia and mortality over a 10 year period [23, 24]. Other response styles, such as tendency to respond using a similar location (e.g. at the extremes, or at a midpoint on a scale) across items has been shown to be related to age [25]. Item non response has been significantly associated with age and cognitive function among older people living in residential care facilities, as well as health status [26, 27].

In the specific context of HRQoL assessment, when used in combination with a digital version of the EQ-5D-5L, eye tracking technology enables an older person’s eye movements to be recorded as they respond, providing information on the distribution of visual attention and information processing [28, 29]. In this way, eye movements can add to the evidence base regarding feasibility by revealing whether participants focus their attention on the worded components of the EQ-5D-5L descriptive system (e.g., dimensions labels, descriptors, response levels) that are important for answering questions relating to their HRQoL without having to rely on their willingness to report whether they have read them or not [29]. Eye tracking may also provide useful information about the worded components of an instrument that the participant has difficulty understanding. For example, a participant may focus for a longer period of time on aspects they are finding difficult to understand, or they may return to key word/s multiple times as they attempt to understand them.

A review of the literature on the application of eye-tracking in health economics and research on cognitive processing in naturalistic tasks is provided in the supplementary information. However, there have been no studies to date which have presented eye-tracking metrics and gaze patterns for completion of self-report PROMs such as the EQ-5D-5L in older people with cognitive impairment.

The main aim of this study was to apply innovative eye tracking technology to provide evidence of the feasibility of older people in residential care with varying levels of cognitive impairment and dementia self-reporting EQ-5D-5L. The results provide insights and evidence to inform decision making about when to seek self-report or proxy-reporting of HRQoL in this population.

## Methods

Details of the data collection procedure for the study are available in supplementary information. Participants recruited were older people living in residential care facilities meeting eligibility criteria including ability to speak and read English fluently, and not currently diagnosed with severe cognitive impairment. Participants were then categorised into one of three cognition subgroups according to National Institute for Health and Care Excellence (NICE) MMSE cognition classifications: no cognitive impairment ≥ 27, mild cognitive impairment 21–26 and moderate cognitive impairment 11–20 [30]. Participants were then assisted to complete a digital self-complete version of the EQ-5D-5L on a Laptop computer with a Tobii Pro Fusion eye tracker attached to record their eye movements [31].

### Data analysis

The eye tracker was controlled using Tobii Pro Eye Tracker Manager software [32] and the data was stored via Tobii Pro Lab software [33]. Tobii Pro Lab’s default Tobii I-VT (Fixation) filter excluded all raw gaze data points that fixated for less than 60 ms or had a velocity of over 30° per second, as used previously in similar studies [28, 34].

**Fig. 1:**
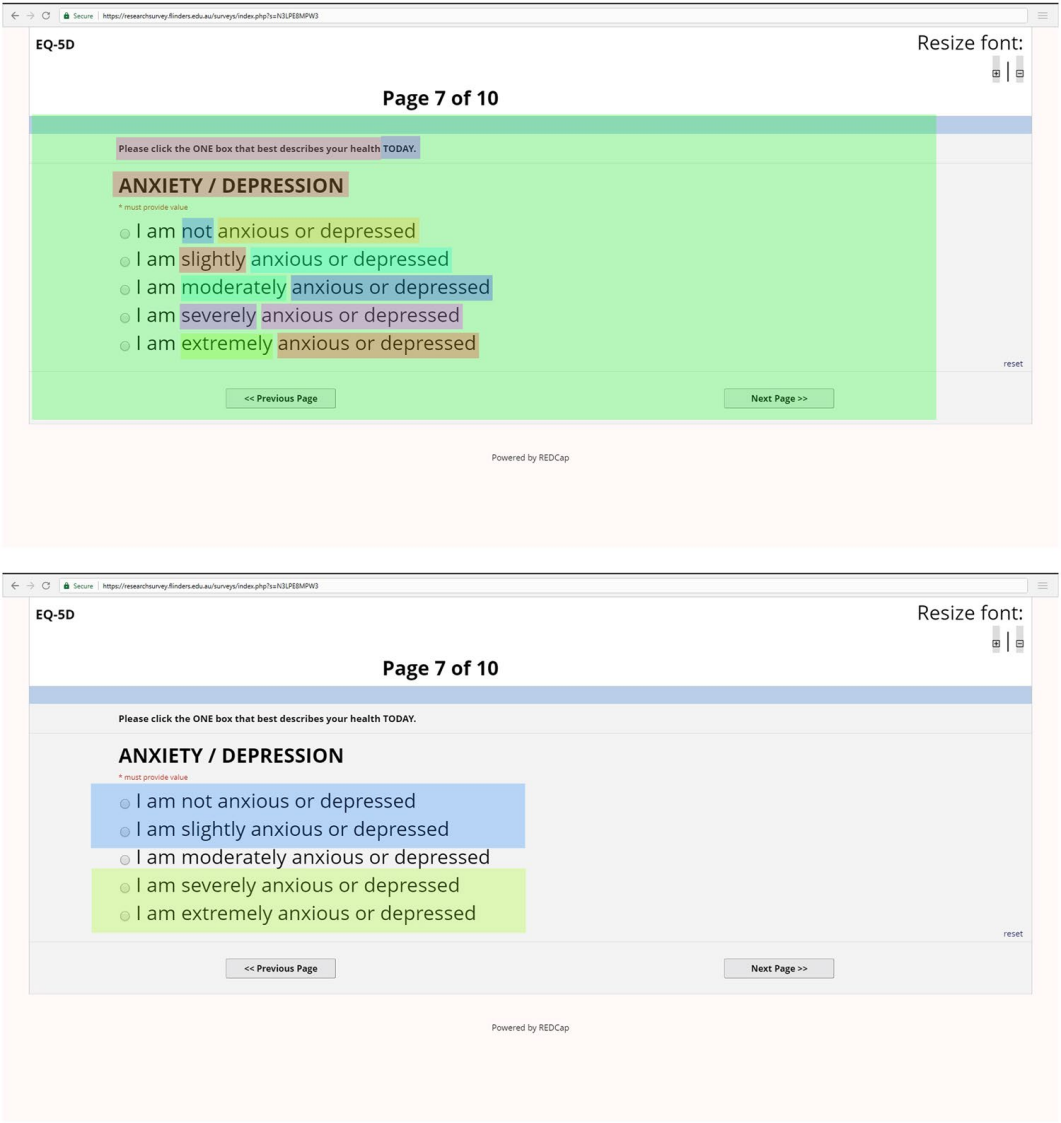
Example relevant areas of interest

For the current study, we chose to focus the eye tracking investigation on the descriptive system itself rather than the VAS, as this is the component most often reported on in quality assessment and economic evaluation. Secondly, due to the small size of the VAS within the official online version of the EQ-5D-5L this component was very difficult for participants to read, and led to high numbers of participants relying on verbal cues from the interviewers, reducing the quality of the data. The decision was therefore made to exclude the eye-tracking data for the VAS. Figure 1 presents an example visual representation of the worded areas of interest (AOI) for the descriptive system. Additional examples of AOI are found in supplementary information.

The degree of attendance (or non-attendance) to AOI representing the important worded components of the descriptive system (dimensions labels e.g., MOBILITY, PERSONAL CARE etc., descriptors e.g., walking around, washing or dressing myself etc. and response levels e.g., no problems slight problems etc.) were calculated for each individual. We also included as AOI the instructions at the top of each dimension (e.g. ‘Please click the ONE box that best describes your health TODAY’) and the time frame that participants were asked to consider in their response (e.g. ‘TODAY). Additionally, an AOI was defined for the entire page for each of the individual five dimensions, which gave information on the number of fixations and saccades within the entire screen. And finally, AOI were defined for the first two (e.g. no problems and slight problems) and bottom two (severe problems and unable/extreme problems) for each of the dimensions. Gaze plots provided a visual representation of the location, and order of focus on the content of each question and associated response levels and were also created for each participant. Similarly, heat maps displaying the focus of visual attention, thereby providing a pictorial representation of the extent of attendance were also created. Only ‘whole fixations’ were included in the analysis i.e. only fixations where the whole fixation was included within the boundaries of the AOI. Where a ‘partial’ fixation was recorded i.e. only partially within the AOI, it was excluded. This was to increase confidence that the fixation being detected by the eye tracker was in fact focusing on the specific AOI.

We also investigated relationship between the response level selected for each dimension, and the response level AOI the participant fixated for the longest amount of time upon. spent the greatest amount of time focusing on. Where the response option participants fixated upon for the longest amount of time was the same response option they selected, a category of “AGREED” (0) was applied, which equates to perfect alignment. Where the response option they fixated upon for the longest amount of time was either immediately prior or subsequent to the response option they chose, a category of ‘±1’ was applied, while ‘±2’ indicates that the response level selected was two levels above or two levels below the response option that the participant fixated upon for the longest amount of time etc. The category “NOTHING” indicates that the participant did not fixate on any of the possible response options before they selected an answer.

All statistical analyses were conducted in R version 4.0.3. The differences in demographic characteristics between the three cognition subgroups were assessed using Chi-Squared Test (for categorical variables) or Kruskal Wallis Test (for non-parametrically distributed continuous variables). The null hypothesis for the Chi-squared test is that there is no difference in the distribution of participants in one categorical variable, according to another categorical variable. The alternative hypothesis for the Chi-squared test is that there is a difference in the distribution of participants into one categorical variable and another categorical variable. The null hypothesis for the Kruskal–Wallis test is that the mean ranks for a certain continuous variable for the groups are the same. The alternative hypothesis is that the mean ranks for a certain continuous variable for at least one of the groups is different to the others. The differences in attendance to the worded components AOI between the cognition subgroups were assessed using Kruskal Wallis Tests. A *p* value of 0.05 or less (two-tailed) was considered statistically significant. Simple effect sizes (eta-squared estimates) were calculated for differences across cognitive impairment subgroups, and were categorised as moderate where they were between 0.06 and less than 0.14, and large where they were 0.14 and above [35].

Several key hypotheses were investigated. Firstly, relating to information processing time and cognition: residents with cognitive impairment would take longer to complete the EQ-5D-5L relative to residents without cognitive impairment in terms of the active time participants spent engaging with the questions and responses included in the instrument. Secondly, relating to concentration upon and attention to the EQ-5D-5L wording AOIs: irrespective of time taken to complete the EQ-5D-5L, residents with cognitive impairment would spend a greater proportion of their time fixated on visual stimuli beyond the key wording of the EQ-5D-5L relative to residents without cognitive impairment. Thirdly, regardless of the level of cognition, for each EQ-5D-5L dimension, residents would spend the most time fixated on the response option they finally chose rather than alternative response options available but not selected. The rationale for these hypotheses, including a discussion of the supporting literature, is available in supplementary information.

A qualitative assessment of the gaze plot videos was also undertaken to give a more detailed assessment of the patterns of movement that participants use when completing the questionnaire. The video for each of the participants was viewed within the Tobii Studio software for each EQ-5D-5L dimension separately. Whether or not a participant exhibited one of eight potential patterns of eye movement was recorded using a binary (Yes/No) response on a researcher developed framework. The eight potential patterns of eye movement were drawn from previous studies in the literature [36–38] finalised via discussion between the researchers.

## Results

A total of 45 older people expressed an interest in participating of whom *n* = 41 (91%) gave informed consent and provided usable data (i.e. calibration component indicated their eyes could be reliably tracked by the system and the inspection of the gaze plot and quality metrics indicated the participant engaged with the online screen and their eyes were within the range to enable collection of reliable data). Table 1 presents a summary of the socio-demographic characteristics of the participating residents. Residents in the no cognitive impairment subgroup were generally younger than those in the mild and moderate cognition subgroups and these differences were statistically significant (*p* < 0.01). Participants with higher levels of cognitive impairment reported relatively better HRQoL on average according to the EQ-5D-5L descriptive system (EQ-5D-5L utility scores) and poorer health according to the EQ-5D VAS, with the differences on the EQ-5D VAS approaching statistical significance (*p* = 0.06).

**Table 1:** Socio-demographic characteristics

Total	Level of cognitive impairment				Test statistic of difference between groups, *p*-value
	None	Mild	Moderate	Total	
	9	20	12	41^a^	
Age					
Mean (SD)	77.6 (6.5)	87.8 (7.95)	90.5 (4.87)	86.4 (8.23)	H(2) = 11.86, *p* ≤ 0.01
Median (25th, 75th percentile)	78.5 (73, 82.5)	89 (80.2, 92.8)	90 (86, 94.5)	87 (80, 93)	
Gender: *n* (%)					
Female	6 (66.67)	11 (55.00)	8 (66.67)	25 (60.98)	*X*^2^ = 1.49, p = 0.48
Male	2 (22.22)	9 (45.00)	3 (25.00)	14 (34.15)	
Education: *n* (%)					
Primary school	1 (11.11)	4 (20.00)	2 (16.67)	7 (17.07)	
Some secondary school	2 (22.22)	4 (20.00)	3 (25.00)	9 (21.95)	*X*^2^ = 3.17, *p* = 0.79
Finished secondary school	1 (11.11)	4 (20.00)	4 (33.33)	9 (21.95)	
Vocational or uni	4 (44.44)	8 (40.00)	2 (16.67)	14 (34.15)	
Time spent living in RACF: *n* (%)					
< 12 m	5 (55.56)	7 (35.00)	4 (33.33)	16 (39.02)	
1-3 year	1 (11.11)	3 (15.00)	4 (33.33)	8 (19.51)	*X*^2^ = 3.90, *p* = 0.42
> 4 year	2 (22.22)	9 (45.00)	3 (25.00)	14 (34.15)	
Birth country: *n* (%)					
Australia	6 (66.67)	14 (70.00)	9 (75.00)	29 (70.73)	
England	1 (11.11)	4 (20.00)	2 (16.67)	7 (17.07)	*X*^2^ = 1.54, *p* = 0.82
Other	1 (11.11)	2 (10.00)	0 (0.00)	3 (7.32)	
Location of RACF: *n* (%)					
Metropolitan	3 (33.33)	6 (30.00)	3 (25.00)	12 (29.27)	*X*^2^ = 0.18, *p* = 0.91
Regional	6 (66.67)	14 (70.00)	9 (75.00)	29 (70.73)	
EQ-5D-5L Utility score:					
Mean (SD)	0.35 (0.47)	0.49 (0.42)	0.65 (0.20)	0.51 (0.39)	*H*(2) = 1.55, *p* = 0.46
Median (25th, 75th percentile)	0.51 (0.00, 0.68)	0.57 (0.08, 0.86)	0.62 (0.49, 0.76)	0.57 (0.19, 0.82)	
EQ-5D-5L VAS score					
Mean (SD)	82.1 (15.6)	77.8 (20.1)	63.1 (22)	74.5 (20.8)	*H*(2) = 5.51, *p* = 0.06
Median (25th, 75th percentile)	85 (75, 90)	78 (70, 95.2)	67.5 (50, 76.2)	75 (65, 90)	
Time taken to complete EQ-5D-5L*: (seconds)					
Mean (SD)	137 (65)	145 (62)	173 (99)	152 (75)	*H*(2) = 0.84, *p* = 0.66
Median (25th, 75th percentile)	108 (97, 184)	132 (93, 192)	136 (112, 218)	130 (98, 192)	

^a^Not all demographic categories sum to 41 because of missing data

*Time recording was measured from the time the Mobility dimension was first displayed on the laptop screen until the time the resident selected their final response for the Anxiety/Depression dimension

*H(2)* Kruskall-Wallis test, with 2 degrees of freedom, *X*^*2*^ chi-squared test, *RACF* residential aged care facility, *SD* standard deviation, *VAS* visual-analogue scale

The 41 participants in the study had an average of 158 saccades (SD 121, ranged 9 to 553) and 290 whole fixations (SD 182, ranged from 11 to 945) included in the analysis. Overall participants engaged with the screen for all of the dimensions, with only one participant who did not have a fixation recorded for the screen for only two of the dimensions (specifically the Usual Activities and Anxiety and Depression dimensions). Participants with cognitive impairment took longer to complete the entire EQ-5D-5L questionnaire on average, based on the time it took the participant to click through the different screens on the laptop to complete the entire questionnaire although these differences were not statistically significant.

Table 2 presents the average amount of time spent (in seconds) focusing on the dimensions overall and the headings/examples by cognition subgroup, i.e. using the information collected by the eye-tracking. For our first hypothesis we expected that residents with cognitive impairment would spend more time overall completing the EQ-5D-5L relative to residents without cognitive impairment. For four out of the five dimensions (with the exception of the usual activities dimension), there was a trend for residents without cognitive impairment to spend less time on average fixated on the dimension text overall, relative to the mild cognitive impairment and moderate cognitive impairment subgroups, although this did not reach statistical significance.

**Table 2:** Average time spent (standard deviation) in seconds focusing on EQ-5D AOIs for dimensions in total and the dimension headings and examples

EQ-5D dimension	Level of cognitive impairment			Test statistic^a^, *p*-value	Effect size^b^
	None	Mild	Moderate		
Mobility	27.1 (17.9)	43.2 (48.1)	37.7 (22.3)	2.1, *p* = 0.35	0.003
Personal care	26.8 (21.6)	47.6 (96.1)	34.0 (26.8)	0.59, *p* = 0.74	− 0.037
Usual activities	36.6 (24.5)	34.4 (31.5)	40.0 (25.3)	0.35, *p* = 0.18	− 0.043
Pain/discomfort	20.4 (11.2)	24.7 (22.0)	25.8 (18.2)	0.15, *p* = 0.93	− 0.049
Anxiety/depression	23.3 (12.7)	25.9 (14.2)	31.3 (29.4)	0.2, *p* = 0.91	− 0.047
Average time spent (standard deviation) in seconds focusing on EQ-5D dimension headings/examples					
Mobility	0.3 (0.4)	0.3 (0.6)	0.2 (0.6)	1.6, *p* = 0.44	− 0.011
Personal care	0.2 (0.2)	0.3 (0.5)	0.4 (1.0)	0.94, *p* = 0.62	− 0.028
Usual activities	1.0 (0.9)	0.2 (0.4)	0.1 (0.2)	9.5, *p* = 0.08	0.197***
Usual activities (examples)	1.4 (1.8)	0.6 (1.5)	0.3 (0.5)	3.0, *p* = 0.22	0.026
Pain/discomfort	0.8 (0.7)	0.3 (0.6)	0.2 (0.3)	6.6,* p* = 0.05	0.121**
Anxiety/depression	1.3 (3.2)	0.3 (0.8)	0.3 (0.5)	0.8,* p* = 0.79	− 0.032

^a^Kruskall–Wallis test, 2 degrees of freedom

^b^Eta-squared estimate

**Indicates a moderate effect size, ***Indicates a large effect size

Our second hypothesis expected that irrespective of the length of completion time overall residents with cognitive impairment would spend a greater proportion of their time fixated on visual stimuli beyond the worded component AOI relative to residents without cognitive impairment. When considering the dimension headings and the example provided for the Usual Activities dimension (presented in Table 2), there was less clear evidence of a trend, and no significant differences in time spent fixating between the cognitive impairment subgroups. There was a trend for participants without cognitive impairment to spend longer focusing on the dimension headings or example for the Usual Activities, Pain and Discomfort, and Anxiety and Depression, but no evidence of a trend for the Mobility or Personal Care dimensions.

Tables 3 and 4 present the average amount of time spent (in seconds) focusing on the dimension descriptors and response levels respectively by cognition subgroup. A graphical summary of the differences in time between the three subgroups is given in supplementary results Figs S2 and S3. None of the differences between the cognition subgroups reached statistical significance across Table 3 or 4. However, upon investigation of Table 3, there is a trend for participants without cognitive impairment to spend longer on average fixated upon the dimension descriptor 1 (ranged from 0.4 to 2.0 s) compared to those in the mild and moderate subgroups (0.2 to 1.1 s). There was no clear evidence of a trend for the descriptors for levels 2 to 5 across the dimensions. There was not clear evidence of those with moderate cognitive impairment fixating for longer than those with mild cognitive impairment, although notably those with moderate cognitive impairment generally spent little time focused on the descriptors consistently across the dimensions (averages ranging from 0.1 to 0.9).

**Table 3:** Average time spent (standard deviation) in seconds focusing on EQ-5D AOIs for the dimension descriptors

EQ-5D descriptor	Level of cognitive impairment			Test statistic^a^, *p*-value	Effect size^b^
	None	Mild	Moderate		
Mobility					
Descriptor 1	1.2 (2.0)	0.4 (0.6)	0.4 (0.6)	2.2, *p* = 0.33	0.005
Descriptor 2	0.4 (0.7)	1.1 (2.2)	0.2 (0.2)	3.9,* p* = 0.14	0.050
Descriptor 3	0.3 (0.6)	0.5 (1.0)	0.3 (0.6)	2.9,* p* = 0.86	0.024
Descriptor 4	0.2 (0.3)	0.3 (0.6)	0.2 (0.5)	2.1,* p* = 0.34	0.003
Descriptor 5	0.4 (0.9)	0.4 (0.6)	0.2 (0.3)	0.47,* p* = 0.79	− 0.040
Personal Care					
Descriptor 1	1.8 (2.6)	0.6 (1.4)	0.5 (1.2)	2.8,* p* = 0.25	0.021
Descriptor 2	1.0 (1.4)	0.4 (0.6)	0.6 (0.9)	0.66,* p* = 0.72	− 0.035
Descriptor 3	0.2 (0.3)	0.9 (1.8)	0.9 (1.7)	0.99,* p* = 0.61	− 0.027
Descriptor 4	0.5 (0.7)	0.6 (1.1)	0.3 (0.6)	0.75,* p* = 0.69	− 0.033
Descriptor 5	0.5 (1.4)	0.4 (1.0)	0.2 (0.4)	1.5,* p* = 0.48	− 0.013
Usual activities					
Descriptor 1	0.4 (0.5)	0.2 (0.2)	0.2 (0.3)	1.8,* p* = 0.40	− 0.005
Descriptor 2	0.4 (0.7)	0.6 (0.8)	0.4 (0.7)	0.78,* p* = 0.68	− 0.032
Descriptor 3	0.4 (0.5)	0.1 (0.3)	0.3 (0.3)	2.0,* p* = 0.37	0.000
Descriptor 4	0.4 (0.5)	0.3 (0.6)	0.4 (0.7)	1.2,* p* = 0.55	− 0.021
Descriptor 5	1.2 (2.7)	0.2 (0.5)	0.1 (0.2)	1.38,* p* = 0.50	− 0.016
Pain/discomfort					
Descriptor 1	2.0 (1.0)	0.6 (1.3)	0.7 (1.1)	2.8,* p* = 0.25	0.021
Descriptor 2	0.7 (0.7)	1.0 (1.2)	0.5 (0.8)	1.9,* p* = 0.38	− 0.003
Descriptor 3	0.6 (0.5)	0.9 (1.3)	0.6 (0.8)	0.34,* p* = 0.84	− 0.044
Descriptor 4	0.3 (0.5)	0.7 (1.1)	0.3 (0.3)	0.9,* p* = 0.64	− 0.029
Descriptor 5	0.9 (0.2)	0.3 (0.5)	0.2 (0.4)	1.2,* p* = 0.56	− 0.021
Anxiety/depression					
Descriptor 1	1.5 (1.6)	0.7 (0.8)	0.8 (1.3)	1.8,* p* = 0.40	− 0.005
Descriptor 2	1.0 (0.9)	1.2 (2.2)	0.5 (0.6)	1.8,* p* = 0.39	− 0.005
Descriptor 3	1.4 (2.0)	0.7 (1.2)	0.3 (0.4)	3.3,* p* = 0.19	0.034
Descriptor 4	0.5 (0.5)	1.0 (1.4)	0.4 (0.7)	0.27,* p* = 0.87	− 0.046
Descriptor 5	0.3 (0.6)	0.5 (0.9)	0.2 (0.4)	0.65,* p* = 0.72	− 0.036

^a^Kruskall–Wallis test, 2 degrees of freedom

^b^Eta-squared estimate

**Table 4:** Average time spent (standard deviation) in seconds focusing on EQ-5D dimension levels

	Level of cognitive impairment			Test statistic^a^, *p*-value	Effect size^b^
Dimension Level	None	Mild	Moderate		
Mobility					
No problems	1.1 (1.3)	0.5 (0.8)	0.7 (0.9)	2.27,* p* = 0.32	0.007
Slight problems	0.7 (0.7)	1.0 (1.6)	0.8 (1.0)	0.19,* p* = 0.91	− 0.048
Moderate problems	0.8 (0.7)	1.1 (1.3)	1.6 (2.7)	0.07,* p* = 0.97	− 0.051
Extreme problems	1.0 (1.0)	0.9 (1.3)	0.7 (1.2)	2.0,* p* = 0.37	0.000
Unable	0.5 (0.7)	0.3 (0.5)	0.3 (0.6)	1.5,* p* = 0.47	− 0.013
Personal care					
No problems	0.1 (0.1)	0.3 (0.5)	1.0 (2.5)	2.4,* p* = 0.30	0.011
Slight problems	0.4 (0.5)	0.5 (0.6)	0.7 (1.0)	1.4,* p* = 0.50	− 0.016
Moderate problems	1.2 (2.1)	0.7 (1.1)	1.4 (2.2)	1.9,* p* = 0.39	− 0.003
Extreme problems	0.8 (1.2)	0.5 (0.9)	0.6 (1.0)	2.8,* p* = 0.24	0.021
Unable	0.3 (0.5)	0.2 (0.5)	0.1 (0.3)	1.0,* p* = 0.61	− 0.026
Usual activities					
No problems	1.2 (1.6)	0.2 (0.5)	0.4 (0.7)	6.4,* p* = 0.06	0.116**
Slight problems	1.1 (1.3)	0.9 (0.9)	0.9 (1.3)	0.72,* p* = 0.70	− 0.034
Moderate problems	1.1 (0.9)	0.8 (1.1)	0.7 (0.8)	2.0,* p* = 0.37	0.000
Extreme problems	1.2 (1.5)	0.6 (0.9)	0.5 ().6)	0.57,* p* = 0.75	− 0.038
Unable	0.3 (0.6)	0.4 (0.6)	0.2 (0.4)	0.33,* p* = 0.85	− 0.044
Pain/discomfort					
No problems	0.1 (0.1)	0.1 (0.1)	0.1 (0.2)	0.76,* p* = 0.68	− 0.033
Slight problems	0.4 (0.5)	0.4 (0.4)	0.2 (0.3)	0.78,* p* = 0.68	− 0.032
Moderate problems	1.2 (2.1)	0.6 (0.8)	0.4 (0.4)	0.94,* p* = 0.62	− 0.028
Extreme problems	0.8 (1.2)	0.4 (0.7)	0.4 (0.6)	0.88,* p* = 0.64	− 0.029
Unable	0.3 (0.5)	0.3 (0.7)	0.1 (0.2)	0.40,* p* = 0.81	− 0.042
Anxiety/depression					
No problems	0.2 (0.3)	0.2 (0.3)	0.4 (1.0)	0.82,* p* = 0.66	− 0.031
Slight problems	0.4 (0.6)	0.4 (0.5)	0.5 (1.1)	0.87,* p* = 0.65	− 0.030
Moderate problems	1.0 (1.5)	0.9 (0.8)	0.5 (1.2)	4.6,* p* = 0.10	0.068**
Extreme problems	0.8 (0.9)	0.6 (0.6)	0.2 (0.3)	2.5,* p* = 0.29	0.013
Unable	0.2 (0.2)	0.5 (1.1)	0.1 (0.3)	1.8,* p* = 0.41	− 0.005

^a^Kruskall–Wallis test, 2 degrees of freedom

^b^Eta-squared estimate

**Indicates a moderate effect size

We also assessed the extent to which participants focused on the “Instructions” AOI and the time duration AOI (i.e. “TODAY”). We identified only few of the participants focused on either of these AOI for any of the dimensions. Only three participants were detected focusing on the “TODAY” AOI. Only 23 participants were detected focusing on the “Instructions” AOI. Only one participant with moderate cognitive impairment focused on the instructions AOI across all of the five dimensions.

**Fig. 2:**
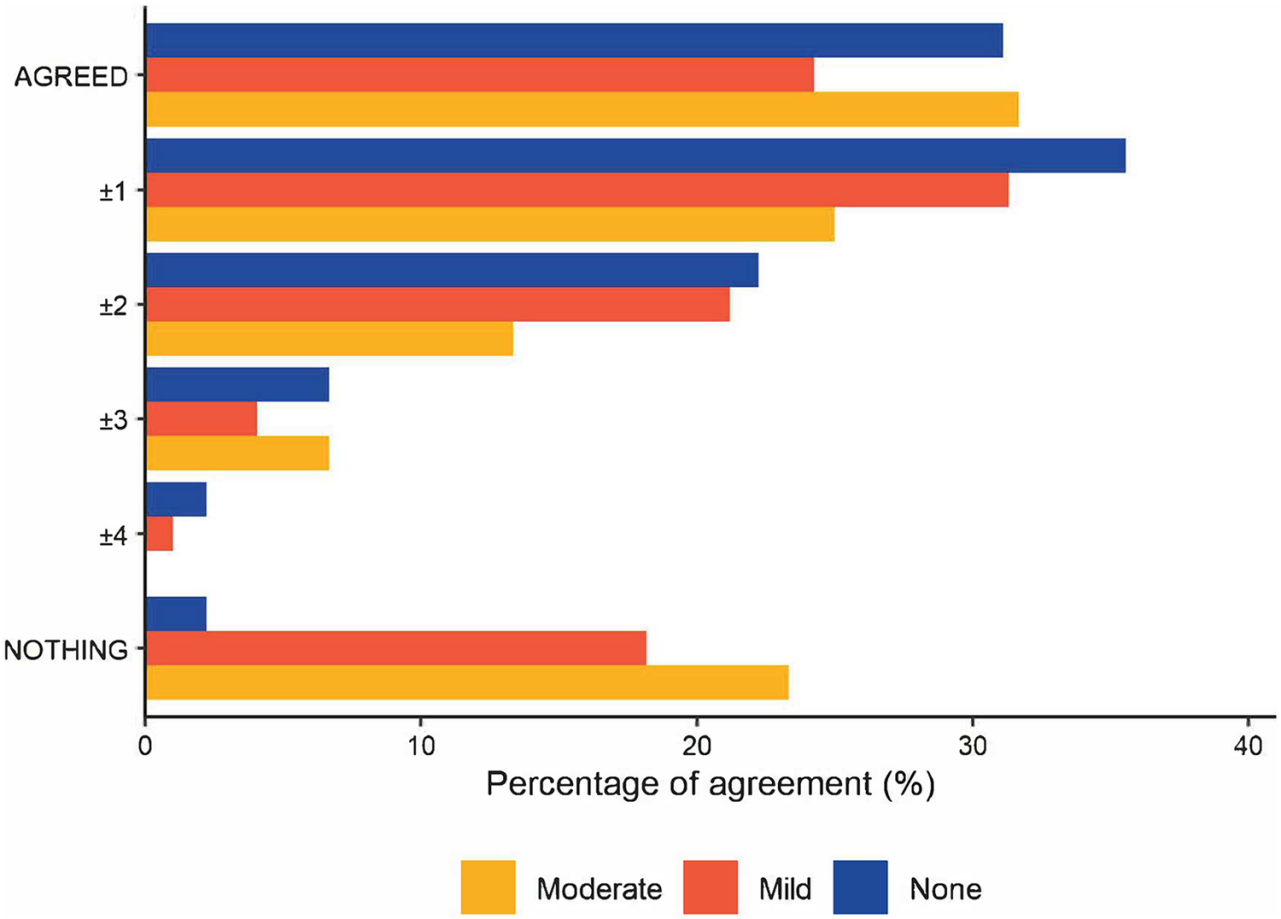
Agreement between time spent focusing on dimension levels and dimension level finally selected. A difference of 4 points between reponse fixated upon and the response chosen is only possible for the highest or lowest possible dimenion levels. For other responses, lower proportions of disagrement (e.g. 3, 2, 1) only be possible. Please note that the figure above presents this data in raw form, and it is not adjusted for whether the level of agreement is possible given the response option chosen by the participant

Figure 2 shows the relationship between the response option the participant fixated upon for the largest amount of time and the response option they finally selected. Our third hypothesis is supported regardless of the level of cognition, i.e. for each dimension, residents tended to spend the most time fixated on the response option they finally chose rather than alternative response options available. Further discussion of the findings across cognitive impairment levels can be found in supplementary information.

Example heat maps (indicating the key areas of the questionnaire participants focused on) and gaze plots (indicating the route the participant eyes took while completing the questionnaire) are provided in Supplementary information. An example is provided for a participant without cognitive impairment, a participant with mild cognitive impairment, and a participant with moderate cognitive impairment. For the heat maps, areas of red indicate they received greater focus from the participants, while areas of green received a lower level of focus. The example from the participant with mild cognitive impairment exhibits some key features. Namely, good coverage of focus across the entire text of the question (i.e. the heat map shows green or red shading of all of the text of the question), and consistent tracking of eyes across the screen targeting key information (i.e. the purple line indicating the route the participant’s eyes took maps to the lines of text well). By comparison the example from the participant with moderate cognitive impairment exhibits different key features. There is little evidence of engagement with the broader text of the questionnaire, with only a few areas shaded green and one area shaded red (the word mobility). There is evidence of a more haphazard approach to finding and reading key information in the gaze plot, with the participant’s eye movements not tracking with the text of the question, but instead seeming to jump to different areas of the screen. Further results including the proportions of participants exhibited specific patterns of eye movement, and who did not record a fixation within an AOI, can be found in supplementary information.

## Discussion

The findings from this exploratory study incorporating eye tracking technology found similar patterns in eye movements between older people without cognitive impairment, as compared to those with mild or moderate cognitive impairment while completing the EQ-5D-5L. There was some evidence that older people with mild or moderate cognitive impairment spent relatively more time completing the EQ-5D-5L than those without cognitive impairment, although completion time differences did not reach statistical significance. By comparison, there was some evidence that older people with mild or moderate cognitive impairment spent less time fixated on component AOIs indicating the text of the questionnaire (for example the dimension headings and examples for the Usual Activities, Pain and Discomfort and Anxiety and Depression dimensions, and Descriptor 1 for all the dimensions) than those without cognitive impairment, which again did not reach statistical significance. The majority of participants fixated for a longer period on the AOI for the dimension level they chose or the level immediately below or above the level they chose, and this was true for all the cognitive impairment subgroups. However, a higher proportion of participants with mild or moderate cognitive impairment failed to fixate on any of the dimension levels when making their selection. A key requirement for assessing the feasibility of self-reported HRQoL assessment with older people in residential care is attendance to the salient components of the EQ-5D-5L descriptive system (e.g., dimensions/domain labels, examples or descriptors, response levels).

This study is also one of few examples of a naturalistic study (i.e. while reading a survey on a computer screen) of eye-tracking in older people with dementia [39]. We found changes in eye tracking metrics and patterns of eye movements similar to those which have been identified in tasks specifically designed to identify cognitive impairment [36]. For example, we identified a more haphazard fixation sequence in our qualitative analysis of the heat maps and gaze plots for those with mild or moderate cognitive impairment compared to those without cognitive impairment. Heat maps for those with cognitive impairment showed less fixation on the text of the questionnaire, and gaze plots displayed eye movements between fixations were more likely to move large distances across the screen and did not follow the text. This could be interpreted as evidence of impairment in executive function controlling involuntary saccades, or a reduced ‘learning effect’ which would usually assist participants to quickly and easily complete the questionnaires which has previously been found in those with cognitive impairment [40, 41]. It is likely that participants with mild or moderate cognitive impairment will need careful consideration of how best to present information within questionnaires to enhance their ability to complete. For example, reducing visual ‘clutter’ on questionnaires, spacing and positioning information to make it easy to find, and not assuming that information is retained from previous questions when moving to subsequent questions may be needed.

Whilst not employing eye tracking technology specifically, several previous studies conducted in Canada, the UK and the US have assessed the feasibility of QoL measurement for older people with mild to moderate cognitive impairment and dementia in residential care settings with generally positive conclusions [42]. The DEMQOL has shown good acceptability, internal consistency, and test–retest reliability in 79 people with mild or moderate dementia (MMSE ≥ 10) and moderate evidence of convergent and discriminant validity [43], while a study in the US found residents classified with moderate or severe cognitive impairment were more likely to experience difficulties with understanding and responding using Likert scale responses in QoL measurement [42].

Very few participants were identified who focused on the instructions for completing the EQ-5D-5L and the recall period (i.e. ‘TODAY’). It is unclear why. The instructions and the recall period are at the top of the screen in very small writing, and thus may not have caught the participant’s attention. The small size of the text may also have reduced the ability of the eye-tracker to detect whether a participant focused on that aspect—for example these may have been recorded as partial fixations and excluded as described in the methods. Evidence available regarding participant interaction with the instructions and the recall period is currently limited. Older hip fracture patients in one study found applying the recall period challenging or considered it not applicable to their situation (as their health changed from day to day) and ignored or reinterpreted it when completing the instrument [44]. Further work assessing comprehension of the recall period and instructions, and how to increase attention to these aspects to ensure the validity of the data collected is needed.

There are several limitations to this study that are important to highlight. Firstly, in relation to the application of the MMSE for determining an older person’s classification to a particular cognition subgroup. This study applied MMSE cognition thresholds published and routinely applied by NICE [45]. However, there are no universally accepted cognition thresholds for determining cognition subgroups using the MMSE and different potential classifications exist [46]. Secondly, the number of participants in this study was limited due to time and resource constraints, including those associated with the COVID-19 pandemic [47]. Although the study found some important differences, a larger sample size may have allowed more precise characterisation of differences between sub-groups. While a larger number of participants would give greater confidence in the findings, we note that studies of small numbers of participants (ranging from 10 to 30 participants) are commonly published in the health services research and survey research literature [34, 48–52]. The nature of eye tracking data also facilitates the collection of a large number of data points within a relatively small sample—i.e. our sample contributed on average 290 whole fixations and 158 saccades per participant to our dataset, resulting in thousands of unique datapoints for analysis. We did not conduct sample size calculations for our study given the exploratory nature of the work. However, future studies could potentially provide more definitive evidence using e.g. information on between group differences from this study, applying power-based sample size calculations available for the relevant tests (for example approaches have been investigated for the Kruskal–Wallis Test [53], in addition to the more traditional methods available [54]). Thirdly, the use of eye-tracking technology may create an inherent bias as the participants are aware that their eye movements are being tracked, possibly changing normal fixation patterns. However, this possibility is arguably reduced with non-wearable eye-tracking devices as applied in this study. We chose to collect data within the residential care facility of the participant rather than transporting participants to a specialist laboratory type environment, which would have provided more consistency and control over the level of lighting, climactic conditions, the type and position of the chair and table, any distractions from sound etc. Instead, we opted for an environment familiar to participants, given known impact of unfamiliar environments on the cognitive load the person experiences and therefore potential impacts on their ability to complete the required tasks in the study [55]. Additionally, no restraints were applied to participants, and so participants were able to move during the data collection. The Tobii Pro Fusion has a level of tolerance for natural movement of participants during data collection. However, we note that the environment for data collection can have an impact on the quality of the data collected [31, 52, 56]. Notably, environment in a room can impact the extent to which a participant’s eye is able to be tracked at all, or the extent to which it can be tracked over time as data collection progresses. We developed a protocol to create the best possible environment within the residential care facility, based on the specific technical specifications of the Tobii Pro Fusion eye tracking system. However, we note that variability in the environment may have impacted upon the data collected for individual participants in ways we are unable to account for in the analysis. We note that some older adults in our sample (*n* = 4) did not provide usable data—either due to inability to complete a successful calibration, or where visual inspection of the gaze plot and quality metrics demonstrated very poor quality data. We acknowledge that for some older people collection of eye-tracking data would not be possible, for example with very thick or multi-focal lenses, or where there is eyelid dysfunction [36].

Whilst undoubtedly providing insightful evidence, this exploratory study has demonstrated that, in and of itself, eye tracking data is unable to conclusively determine the feasibility (or otherwise) of self-reported HRQoL assessments using the EQ-5D-5L with older people with varying levels of cognitive impairment in residential care. Consequently, the use of eye tracking data is unlikely to be definitive in isolation to generate guidance on the appropriate use of self-assessment versus proxy-assessment. Eye-tracking, however, can be used alongside traditional methods to assess the feasibility and acceptability of self-report. The research team is engaged in additional research including qualitative think aloud whereby the resident is prompted to explain their understanding and justify their responses to each EQ-5D dimension. This approach provides further evidence which, when used alongside eye tracking evidence, may facilitate a more comprehensive assessment of feasibility. While our findings are in the context of the EQ-5D-5L, we believe that the general patterns are applicable to other instruments. It may be that, in larger instruments with less standardisation of wording across attributes, difficulty in completion may begin at more mild levels of cognitive impairment.

## Conclusions

In conclusion, this exploratory study has provided for the first time information on the eye movements of older adults with a range of cognitive abilities when self-completing a HRQoL instrument (the EQ-5D-5L). We have identified patterns of eye movements which signify the feasibility of self-reporting the EQ-5D-5L among older adults without cognitive impairment and with mild cognitive impairment. As expected, there was some evidence of patterns of eye movements indicative of difficulty obtaining and processing information for older adults with moderate cognitive impairment. Future research should focus on combining research from eye-tracking with other qualitative methods to produce a robust evidence base to support decisions about the nexus between self-report and proxy report in this population.

### Supplementary Information

Below is the link to the electronic supplementary material.

Supplementary file1 (DOCX 25 kb)

Supplementary file2 (DOCX 2068 kb)

## Data Availability

The datasets generated and analysed during this study are available from the corresponding author on request.

## Abbreviations

EQ-5D-5L: EuroQoL five dimension five level
HRQoL: Health-related quality of life
MMSE: Mini-mental state examination
QoL: Quality of life
RACF: Residential aged care facility
