# Supplementary material for: Feasibility of self-reported health related quality of life assessment with older people in residential care: insights from the application of eye tracking technology
Source: Qual Life Res. 2023 Jul 20;32(12):3557–69. doi: 10.1007/s11136-023-03488-w (PMC10624716; doi:10.1007/s11136-023-03488-w)
Supplement: Supplementary file 1 — Supplementary file1 (DOCX 25 kb) [file 11136_2023_3488_MOESM1_ESM.docx]

**Supplementary Literature and Methodology**

**Background Information on the EQ-5D-5L**

The EQ-5D-5L comprises two main components [22]. Firstly, a descriptive system for measuring HRQoL, comprises five HRQoL dimensions (mobility, self-care, usual activities, pain/discomfort and anxiety/depression) with five possible response levels reflecting increasing levels of impairment. Secondly, there is a visual analogue scale (VAS), ranging from 0 (worst imaginable health) to 100 (best imaginable health). The descriptive system has a variety of accompanying preference-based scoring algorithms or value sets (often based on general population samples from specified countries) for scoring health states defined by the EQ-5D-5L on the full health (1) to dead (0) utility scale required for the calculation of quality adjusted life years (QALYs) for economic evaluation [19].

**Literature on Eye Tracking in Health Economics and Cognitive Processing Research**

In the health economics literature, several previous studies have applied eye tracking technology to investigate the cognitive processing strategies adopted by health and social care consumers with no cognitive impairment in making choices using discrete choice experiment methodology [1-3]. Wang and colleagues applied eye-tracking technology to investigate information processing approaches adopted by older people with and without cognitive impairment in determining discrete choice experiment (DCE) generated preferences for health states defined by the EQ-5D-5L instrument [4]. This study found that non-attendance to key DCE attributes (comprising key dimensions labels, descriptors and response levels from the EQ-5D-5L) was higher for participants with mild cognitive impairment relative to those with no cognitive impairment, although these differences were generally not statistically significant.

In addition, there is a growing body of evidence that significant changes in eye tracking metrics can be detected among those with and without mild cognitive impairment and dementia, or even between those with mild cognitive impairment versus diagnosed dementia [5; 6]. Studies of eye tracking in this population have focused on two forms of study – studies using tasks specifically developed to test for subtle changes in cognitive status, and studies of participants performing ‘naturalistic’ tasks, such as walking around a room, reading a computer screen (as was the case in this study), or performing basic activities of daily living [6]. Studies utilising naturalistic tasks are fewer in number. However, it is now clear that eye-tracking when used in combination with specifically developed tasks can be successful in identifying people with mild cognitive impairment, and those with mild cognitive impairment at risk of developing dementia in the future [7; 8]. For example, errors on the Antisaccade Task (where participants are asked to inhibit a response towards a stimulus and voluntarily shift their eyes in the opposite direction) are correlated with executive function decline as well as physical changes in the brain associated with cognitive decline and dementia [6]. Conversely, a Prosaccade is a movement towards a stimulus when asked to focus on it. There are two types of prosaccades which are important to consider in this context. Voluntary saccades are under the control of the participant and are deliberate movements of the eye towards or away from a stimulus in response to instructions. By contrast, visually guided saccades are involuntary eye movements as a result of an event in the field of vision e.g. the appearance of a new image. Voluntary saccades are thought to recruit high-level executive functions to a greater extent than visually guided saccades. Voluntary eye movements involve participants identifying the action they wish to perform, suppressing involuntary actions that do not achieve the action or goal they wish to perform, and then performing the desired action [8]. This is in contrast to visually guided saccades which do involve such steps. There is some evidence that prosaccades are not impaired or are less impaired in people with cognitive impairment than antisaccade movements [5]. Researchers have hypothesised that the impairment to control of antisaccade movements occurs as these movements require higher order executive function control as they involve inhibition of a visually guided saccade as well as implementation of a voluntary saccade.

Another specifically developed test for cognitive impairment is the Visual Paired Comparison Task [5]. Designed to test episodic memory it has two stages. Firstly an image is presented, usually on a computer screen. After a short delay, the same image is presented side-by-side with a new image. The amount of time the participant spends exploring each image is measured. Participants without cognitive impairment would be expected to spend more time focusing on the new image, due to novelty preference. However, participants with impaired memory were found to spend greater amounts of time looking at the previously presented image, and this was found to discriminate between participants with and without mild cognitive impairment, as well as to be predictive of development of mild cognitive impairment in the future, or of the development of Alzheimer’s disease among those with mild cognitive impairment.

Other key considerations in eye-tracking analysis are the fixation sequence (i.e. where the participant focused throughout the data collection period) and revisits (i.e. whether participants return to look again at locations of the screen they had focused on before) [9; 10]. The areas participants look at first are important to consider, as they often reflect their areas the participant is most interested in, as well as parts of the stimulus which stand out due to formatting and display of information. When making a decision between multiple options presented as a stimulus, often participants will focus last on the option they finally choose. Revisits on the other hand, may occur when a participant finds an image pleasing, or confusing. Other studies have used free-recall tests of images (either related to each other or unrelated) to understand to what extent encoding (i.e. the formation of memories) is impacted by age and cognitive impairment, as compared to the retrieval of information [11]. Suzin et al. [11] identified significantly lower free-recall of images in healthy older participants, and participants with cognitive impairment. For the younger adults, presentation of images which were related to each other facilitated greater free-recall as compared to completely unrelated images. For participants with MCI, however there was no impact of the relatedness of the images. Analysis of eye-tracking found that younger participants demonstrated a semantic sequence of fixations (i.e. where related objects were viewed next to each other), versus a sequency where they scanned across the rows. Younger participants also showed a ‘learning effect’ in that they showed an increasing tendency to use semantic sequences throughout the trials, while the older people and participants with MCI they did not show this learning effect.

**Supplementary Methodology**

***Study Design***

Residential care facilities that expressed an interest in study participation were visited by two members of the researcher team (MC and KL) who delivered a presentation outlining the details of the study. Residents were given an information sheet and referred to their facility manager if they were interested in participating. To be eligible residents had to be 65 years or older, able to speak and read English fluently, not currently diagnosed with any known eye conditions that would preclude the collection of eye tracking data (e.g., nystagmus, blindness or cataract), not currently diagnosed with severe cognitive impairment and have a close family member who would be able to participate in the study. Written informed consent was obtained from each study participant prior to commencing the study which was conducted as a semi-structured face-to-face interview.

***Interview procedure***

Each interview was conducted in the resident’s room or in another space nominated by the resident. Firstly, a widely used and accepted clinical indicator of cognitive impairment, the Mini Mental State Examination (MMSE), was administered with the person to determine their eligibility to progress with the interview (Score >10) [33].

A laptop computer was positioned in front of the participant with a Tobii Pro Fusion eye tracker attached to the base of the screen and calibrated to the participant’s eyes. Participants were asked to complete a digital self-complete version of the EQ-5D-5L. Participants were advised that their eyes would be tracked while completing the questionnaires on the computer, and that this was to understand how they filled out the questionnaire. Participants were positioned according to a protocol developed to collect the best quality of eye-tracking data according to technical specifications, but within the limitations of the participant group (who are often physically frail in addition to any cognitive impairments) [35]. Ideally participants were seated within their own room usually, with overhead lighting turned on but away from direct sunlight from windows. They were seated in a stable stationary chair, with the computer on a height adjustable table around 60 cm in front of them. The participants were asked to remain relatively still, however the eye-tracker has the ability to account for normal levels of head movement during data collection. Participants were not restrained, and a chin rest was not used, due to the potential distress this could cause in our sample population. At times the participants would break eye contact with the screen to talk to the researcher. Where this occurred, the researcher would gently direct the participant back to the task on the screen. To overcome variations in participants’ familiarity using a computer, the researcher operated the computer mouse and responded to the resident’s responses and instructions to move through the questionnaire. Following completion of the EQ-5D-5L, participants were asked a series of sociodemographic questions which included their age, gender, country of birth and length of time spent living in the residential care facility.

***Rationale for Hypotheses Tested***

The rationale for the first hypothesis (i.e. “residents with cognitive impairment would take longer to complete the EQ-5D-5L relative to residents without cognitive impairment in terms of the active time participants spent engaging with the questions and responses included in the instrument”) was drawn from available evidence that people with mild cognitive impairment and dementia experience declines in not only their memory (which is most commonly associated with the condition) but also declines in other aspects of cognition such as attention, visual-spatial abilities, executive functioning skills and information processing speed [12-14]. Particularly declines in executive functioning skills, such as inhibition, working memory, and cognitive flexibility, would impair the ability of participants to prioritise the EQ-5D-5L task, avoid getting distracted by other stimuli while undertaking the task, and retain and work with information derived from reading the questionnaire in combination with information about their own current health state. The net result of these declines in executive function would be to increase the time taken the process the information in the questionnaire and formulate a response, increasing total time taken to complete the questionnaire. Importantly, we would be able to undertake this analysis without relying on participants providing this information themselves or interviewer judgement, although it should be noted that simple recording of response time in an online survey also achieves this aim.

The rationale for the secondly hypothesis (i.e. that “irrespective of time taken to complete the EQ-5D-5L, residents with cognitive impairment would spend a greater proportion of their time fixated on visual stimuli beyond the key wording of the EQ-5D-5L relative to residents without cognitive impairment”, was based on evidence that that people with dementia experience impairment to their executive functioning [4], which reduces inhibition which would usually work to maintain a person’s focus on a task to achieve completion. Therefore, a person with dementia would be more distracted, and fixate on parts of the screen not used to complete the EQ-5D-5L than a person without dementia.

The rationale for the third hypothesis, (i.e. that “regardless of the level of cognition, for each EQ-5D-5L dimension, residents would spend the most time fixated on the response option they finally chose rather than alternative response options available but not selected”) was based on the assumption of the ‘eye-mind hypothesis’ which assumes that information processing occurs during the fixation [15]. We expected that participants would spend the majority of their time focusing on the option that they final chose or the immediately subsequent option [16]. This is based on the concept of ‘satisficing’ proposed by Krosnick, which suggests that rather than looking at all the information in a survey in detail, participants would take shortcuts [7]. Galesic et al. [16] found empirical evidence of ‘satisficing’ using eye-tracking among predominantly college student respondents. As an example of a strategies participants could use, is to move through the response options sequentially until they come to an option that is a ‘good enough’ reflection of their true response. Participants then do not read the remaining response options, instead focusing on and choosing the ‘good enough’ response option. The net result of this strategy would be that participants spend more time focusing the response option they finally chose or those immediately before or after it. We expected that this approach would be used across all cognitive impairment groups, indicating that satisficing is part of normal cognitive processing, rather than purely associated with impaired cognition.

**References**

1. Spinks, J., & Mortimer, D. (2016). Lost in the crowd? Using eye-tracking to investigate the effect of complexity on attribute non-attendance in discrete choice experiments. *BMC Med Inform Decis Mak*, 16, 14.

2. Ryan, M., Krucien, N., & Hermens, F. (2017). The eyes have it: Using eye tracking to inform information processing strategies in multi-attributes choices. *Health Econ*.

3. Vass, C., Rigby, D., Tate, K., Stewart, A., & Payne, K. (2018). An Exploratory Application of Eye-Tracking Methods in a Discrete Choice Experiment. *Med Decis Making*, 38(6), 658-672.

4. Wang, K., Barr, C., Norman, R., George, S., Whitehead, C., & Ratcliffe, J. (2021). Using Eye-Tracking Technology with Older People in Memory Clinics to Investigate the Impact of Mild Cognitive Impairment on Choices for EQ-5D-5L Health States Preferences. *Appl Health Econ Health Policy*, 19(1), 111-121.

5. Bueno, A. P. A., Sato, J. R., & Hornberger, M. (2019). Eye tracking - The overlooked method to measure cognition in neurodegeneration? *Neuropsychologia*, 133, 107191.

6. Seligman, S. C., & Giovannetti, T. (2015). The Potential Utility of Eye Movements in the Detection and Characterization of Everyday Functional Difficulties in Mild Cognitive Impairment. *Neuropsychol Rev*, 25(2), 199-215.

7. Krosnick, J. A. (1991). Response strategies for coping with the cognitive demands of attitude measures in surveys. *Applied Cognitive Psychology*, 5(3), 213-236.

8. Opwonya, J., Wang, C., Jang, K. M., Lee, K., Kim, J. I., & Kim, J. U. (2022). Inhibitory Control of Saccadic Eye Movements and Cognitive Impairment in Mild Cognitive Impairment. *Front Aging Neurosci*, 14, 871432.

9. Djamasbi, S., & Hall-Phillips, A. (2014). Visual Search. In *Eye Tracking in User Experience Design* (pp. 27-45).

10. Colter, A., & Summers, K. (2014). Low Literacy Users. In *Eye Tracking in User Experience Design* (pp. 331-348).

11. Suzin, G., Ravona-Springer, R., Ash, E. L., Davelaar, E. J., & Usher, M. (2019). Differences in Semantic Memory Encoding Strategies in Young, Healthy Old and MCI Patients. *Front Aging Neurosci*, 11, 306.

12. Guarino, A., Forte, G., Giovannoli, J., & Casagrande, M. (2020). Executive functions in the elderly with mild cognitive impairment: a systematic review on motor and cognitive inhibition, conflict control and cognitive flexibility. *Aging Ment Health*, 24(7), 1028-1045.

13. de Jager, C. A. (2004). Changes over time in memory, processing speed and clock drawing tests help to discriminate between vascular cognitive impairment, mild cognitive impairment and Alzheimer's disease. *Neurological Research*, 26(5), 481-487.

14. Vivot, A., Glymour, M. M., Tzourio, C., Amouyel, P., Chene, G., & Dufouil, C. (2015). Association of Alzheimer's related genotypes with cognitive decline in multiple domains: results from the Three-City Dijon study. *Mol Psychiatry*, 20(10), 1173-1178.

15. Rigby, D., Vass, C., & Payne, K. (2020). Opening the 'Black Box': An Overview of Methods to Investigate the Decision-Making Process in Choice-Based Surveys. *Patient*, 13(1), 31-41.

16. Galesic, M., Tourangeau, R., Couper, M. P., & Conrad, F. G. (2008). Eye-Tracking Data: New Insights on Response Order Effects and Other Cognitive Shortcuts in Survey Responding. *Public Opin Q*, 72(5), 892-913.
