# Supplementary material for: Feasibility of self-reported health related quality of life assessment with older people in residential care: insights from the application of eye tracking technology
Source: Qual Life Res. 2023 Jul 20;32(12):3557–69. doi: 10.1007/s11136-023-03488-w (PMC10624716; doi:10.1007/s11136-023-03488-w)
Supplement: Supplementary file 2 — Supplementary file2 (DOCX 2068 kb) [file 11136_2023_3488_MOESM2_ESM.docx]

**Supplementary Files Figure 1: Example areas of interest for remaining dimensions**


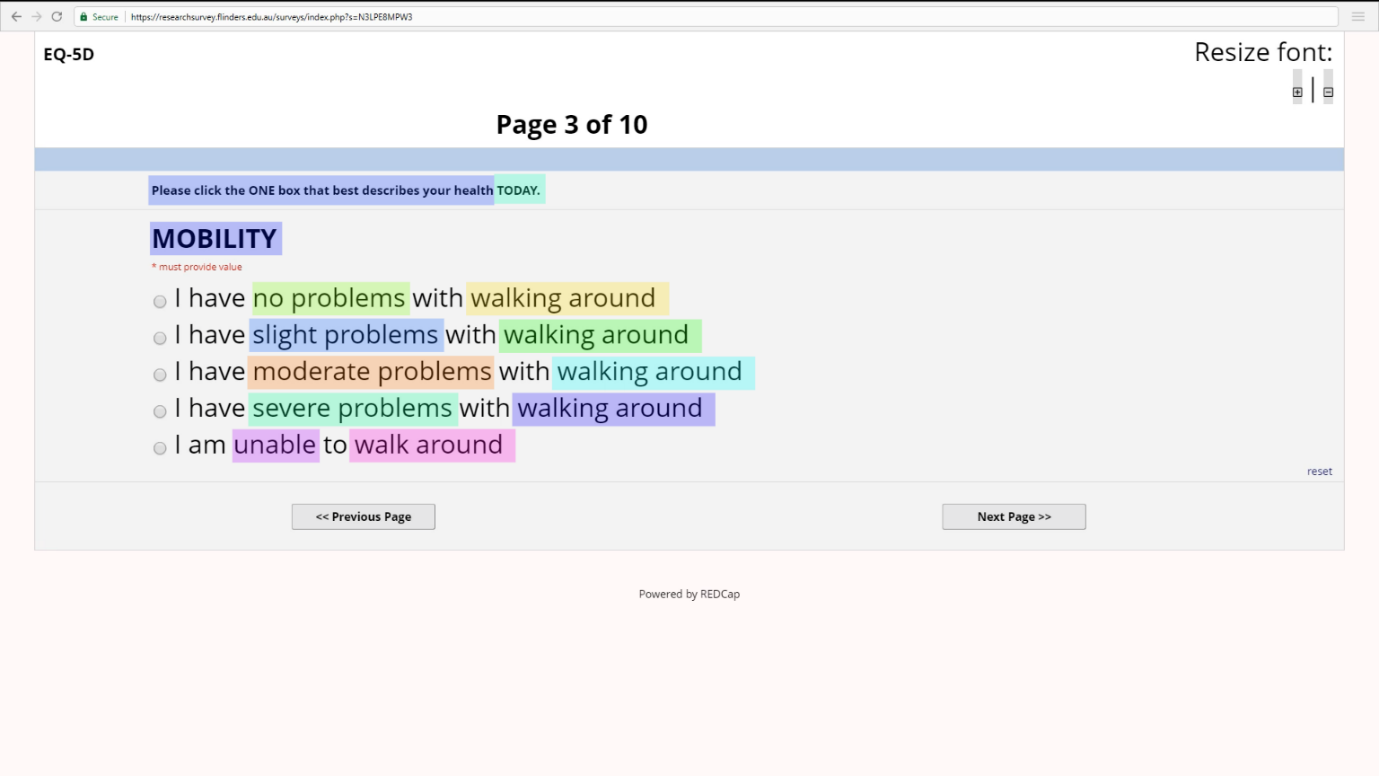


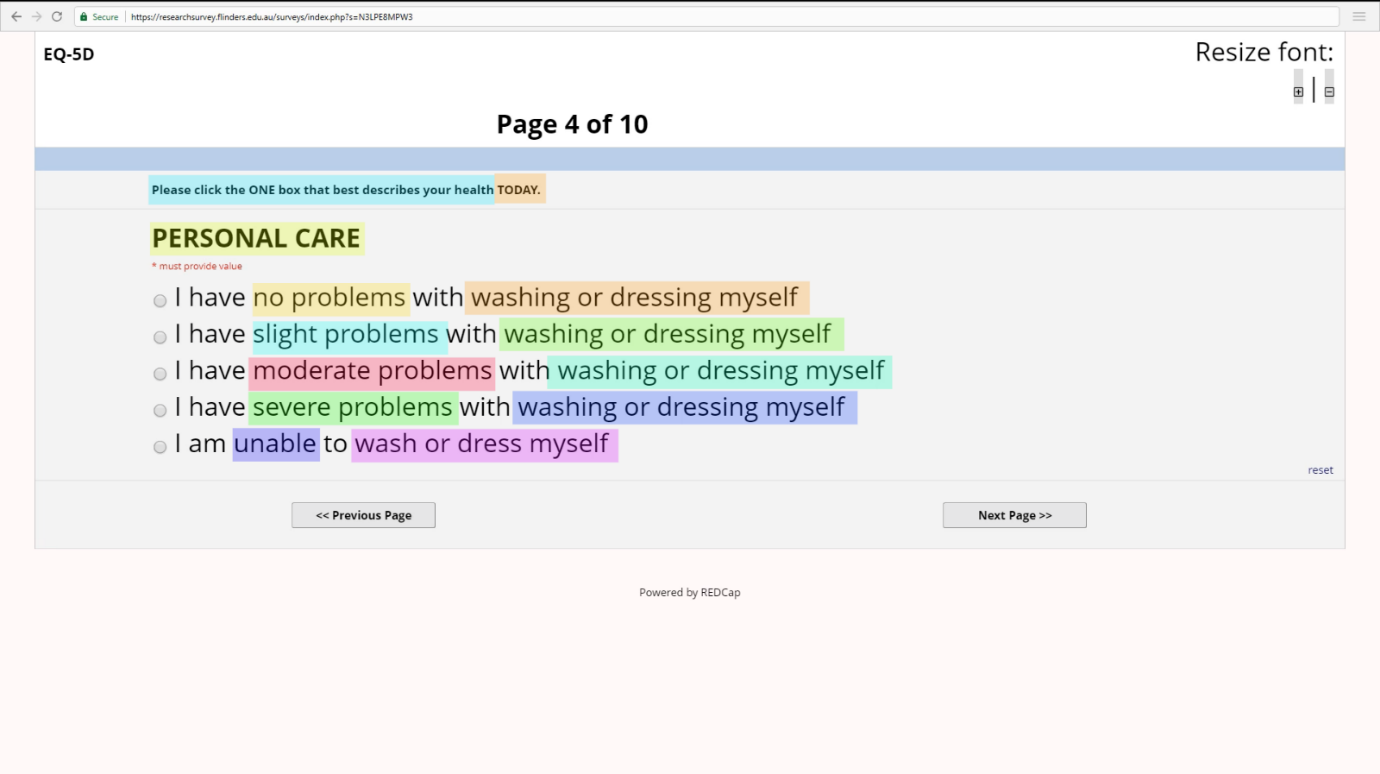


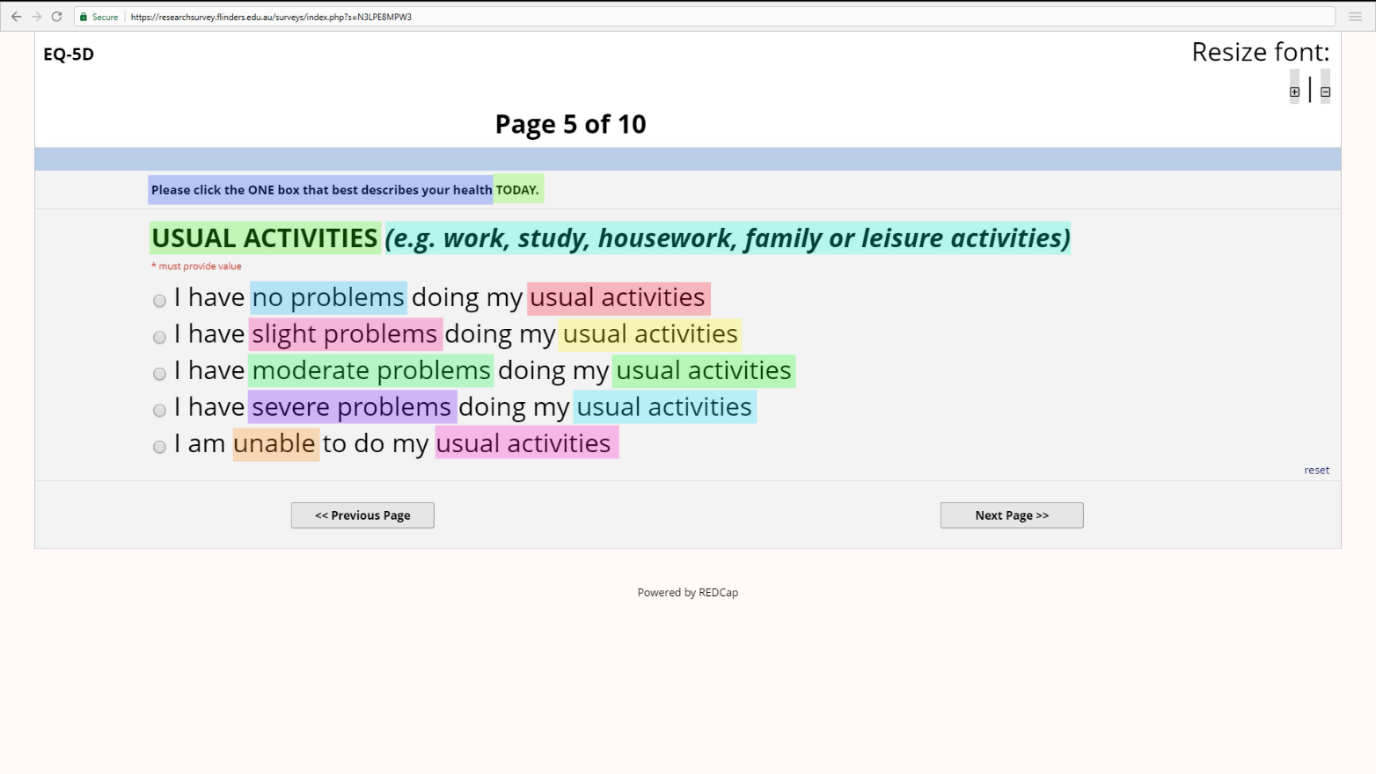


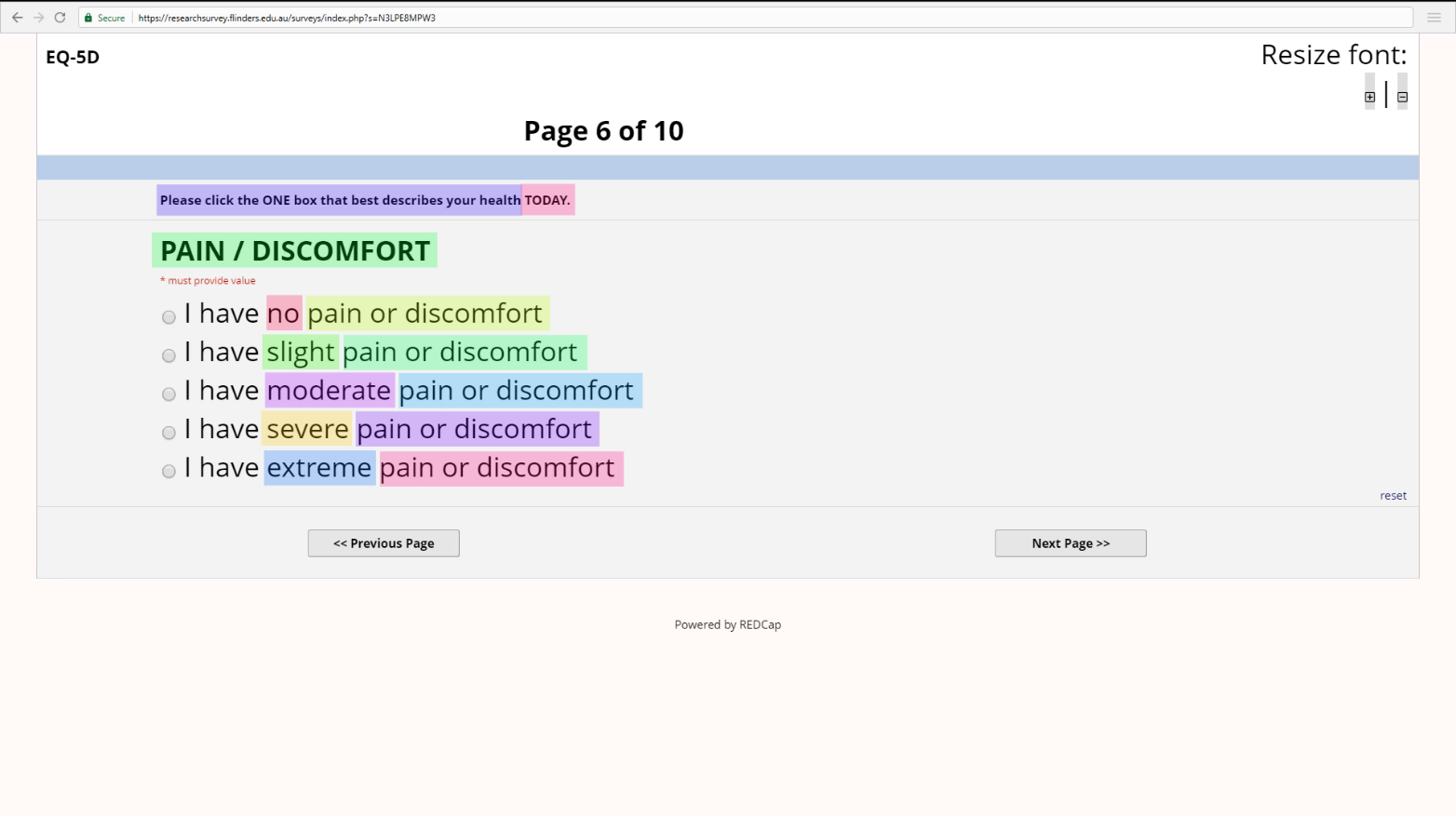

**Supplementary Figure 2:** Visual summary of the differences in averages in fixation time on the dimension descriptors between the three cognitive impairment subgroups.

NB: Green shading indicates a positive value (e.g. average time spent by the subgroup with greater cognitive impairment is larger than the time spent by the subgroup with less cognitive impairment), Red indicates a negative value (e.g. average time average time spent by the subgroup with greater cognitive impairment is smaller than the time spent by the subgroup with less cognitive impairment), and yellow shading indicates no difference. Please note that none of the differences reached statistical significance.

**Supplementary Figure 3:** Visual summary of the differences in averages in fixation time on the dimension levels between the three cognitive impairment subgroups.

NB: Green shading indicates a positive value (e.g. average time spent by the subgroup with greater cognitive impairment is larger than the time spent by the subgroup with less cognitive impairment), Red indicates a negative value (e.g. average time average time spent by the subgroup with greater cognitive impairment is smaller than the time spent by the subgroup with less cognitive impairment), and yellow shading indicates no difference. Please note that none of the differences reached statistical significance.

**Supplementary Information: Example heat maps**

Participant ID: 013 75 years old; male; no cognitive impairment


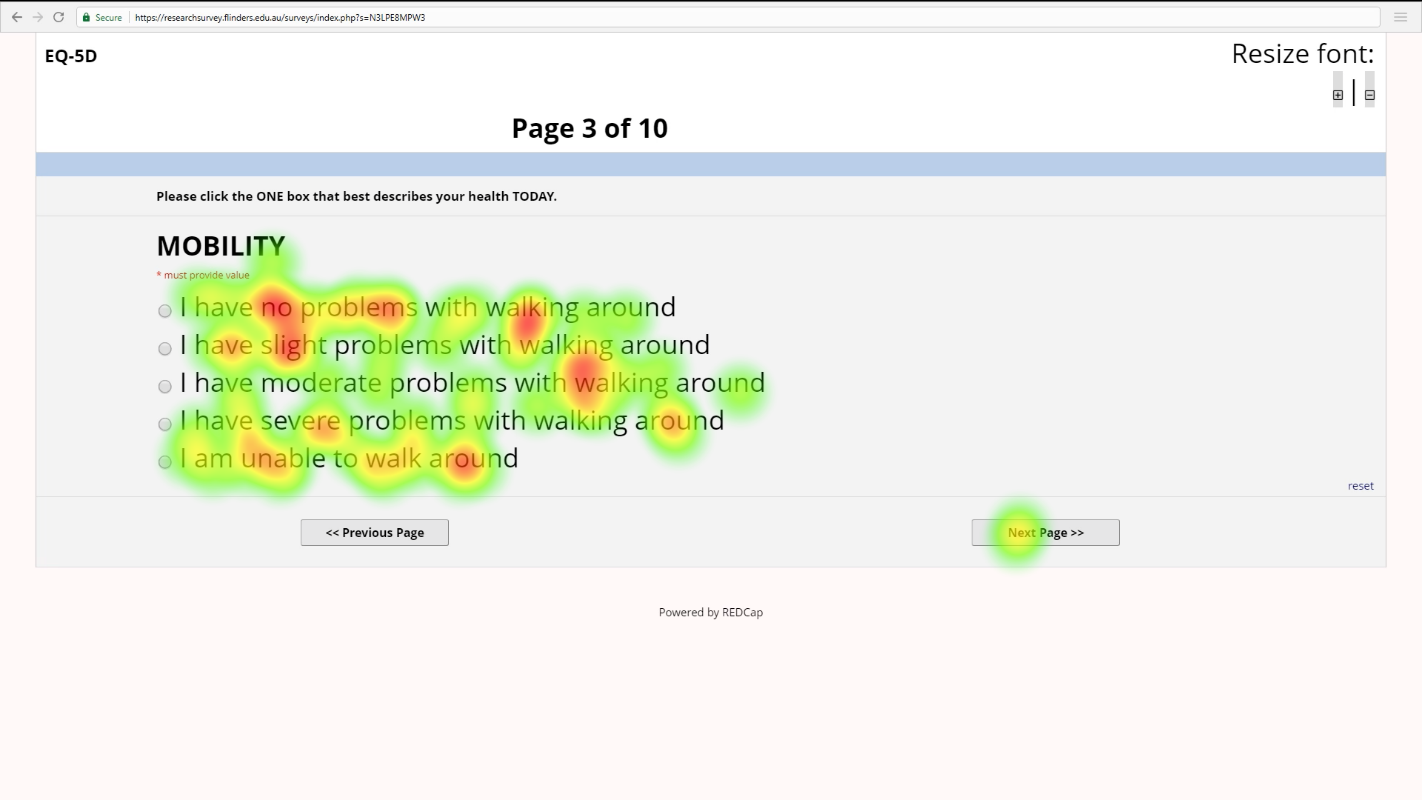


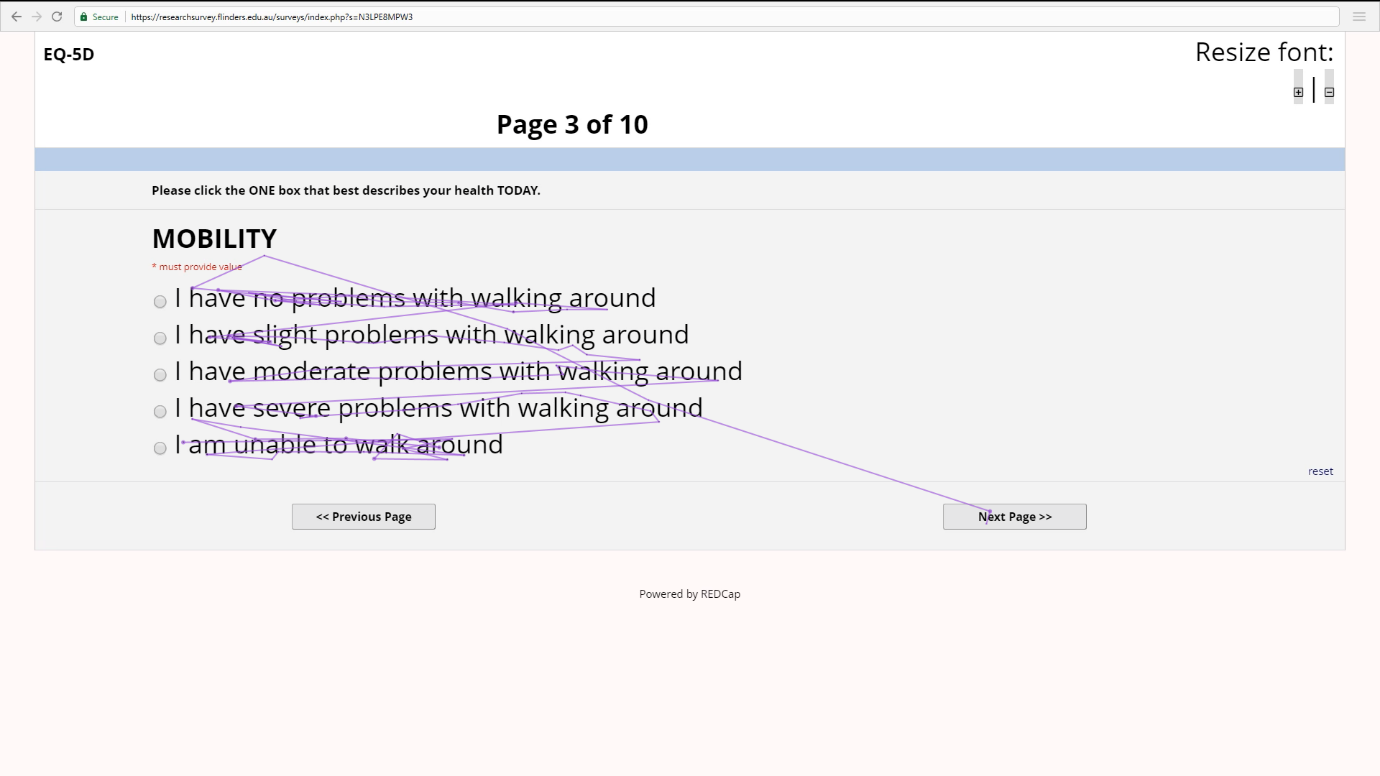


Participant ID 06: 90 years old; male; mild cognitive impairment


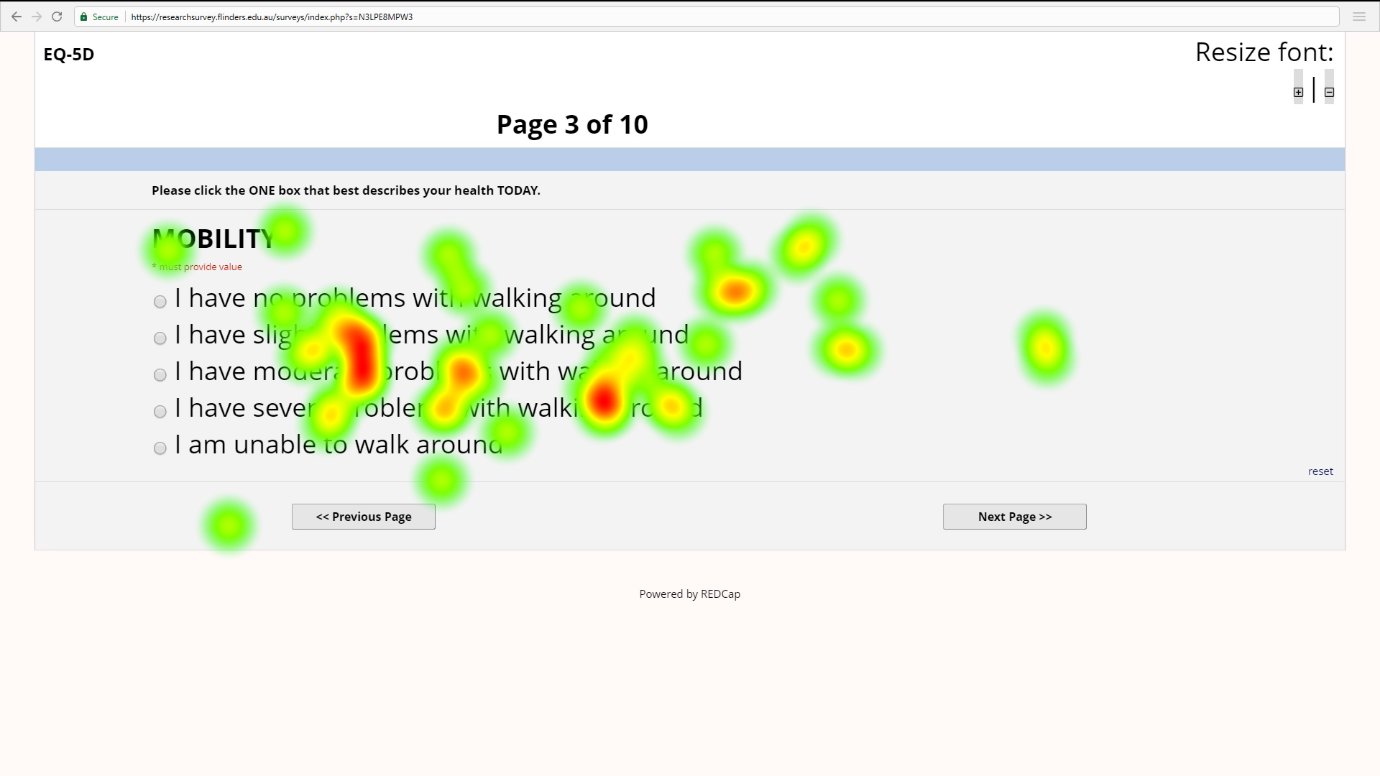


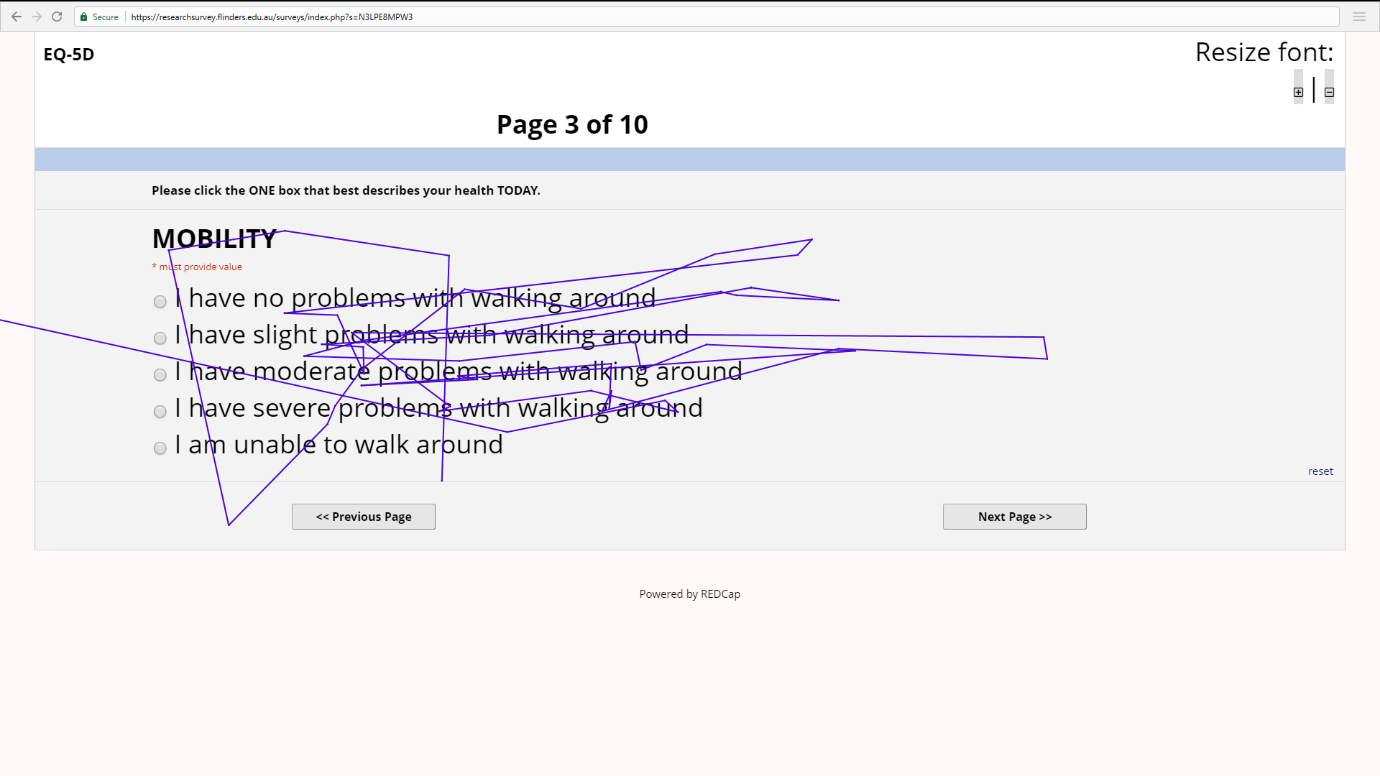


Participant ID 022: 94 years old; male; moderate cognitive impairment


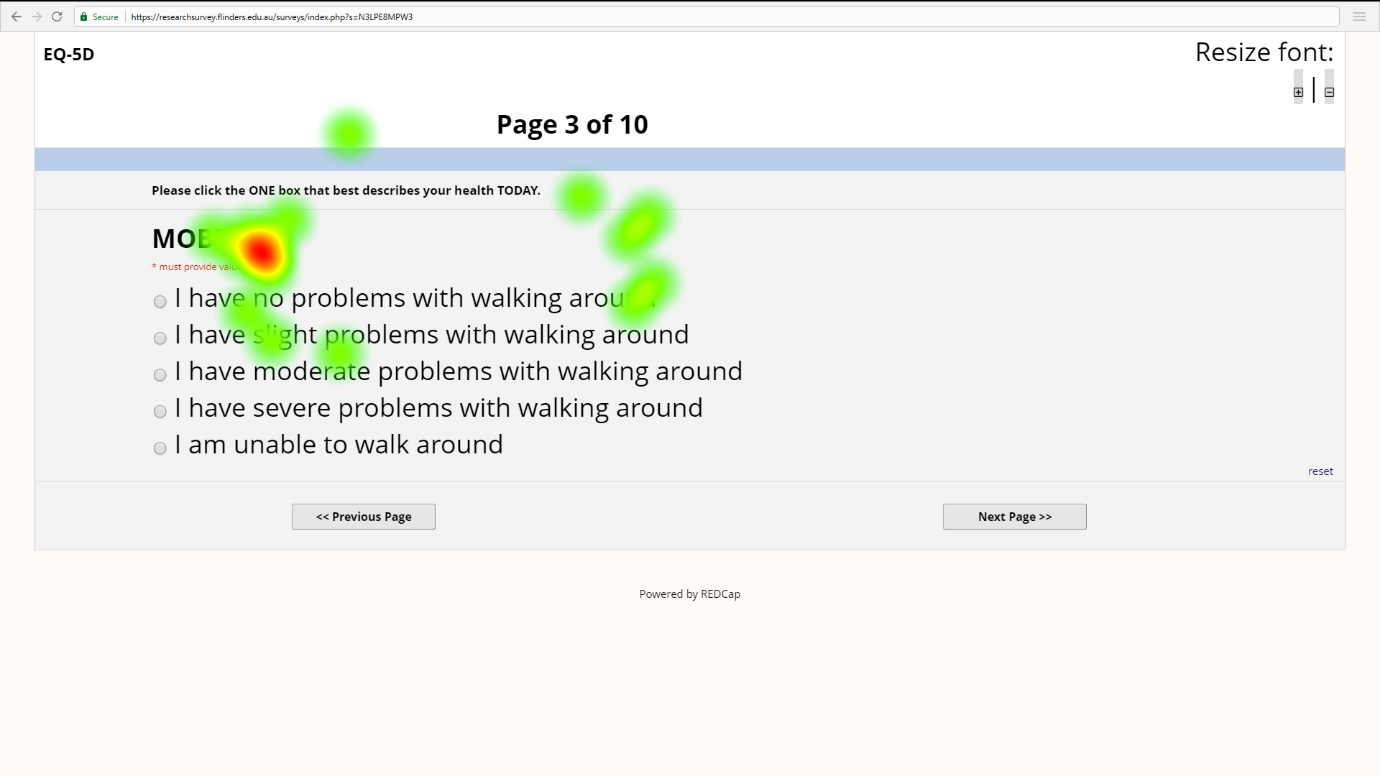


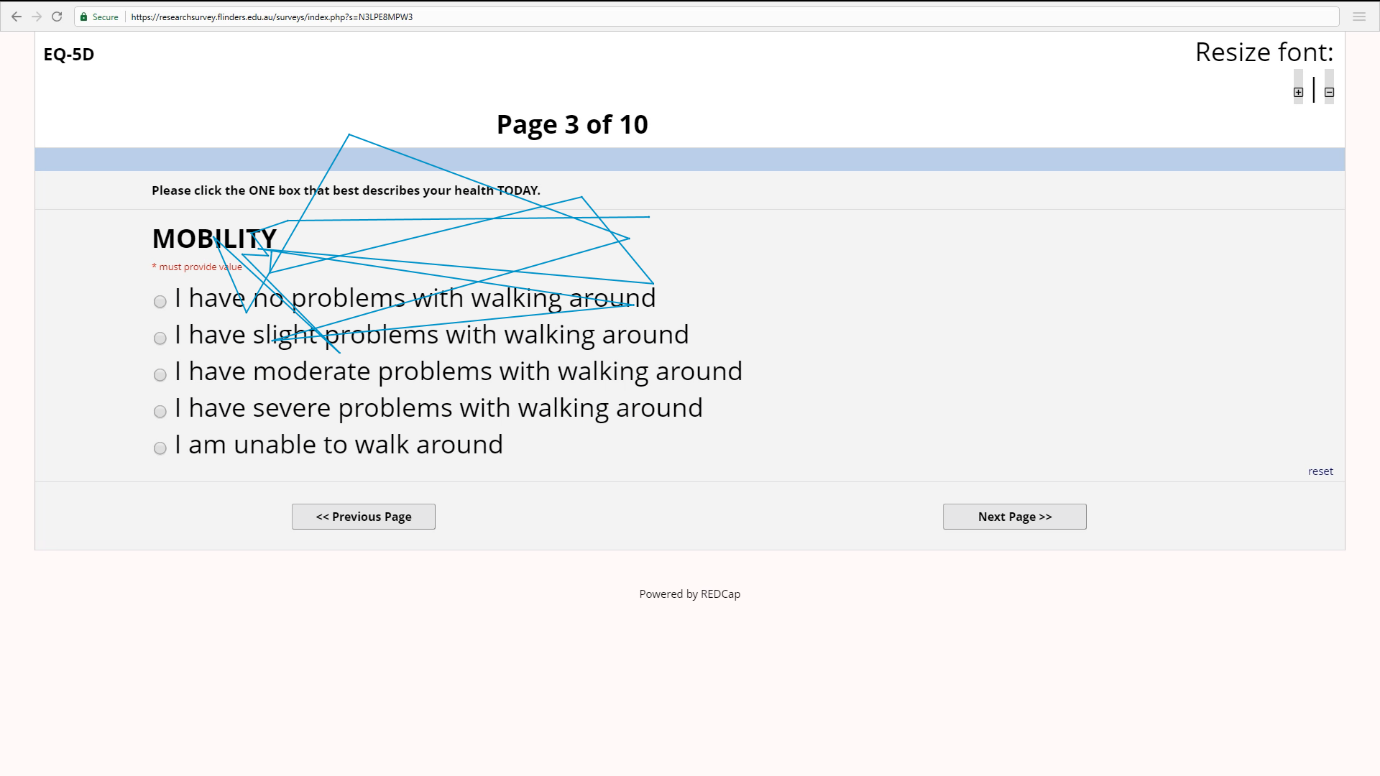


**Supplementary Results**

Some important differences were evident across cognition subgroups presented in Figure 2. Participants with no cognitive impairment were more likely to fixate upon the response option they selected or the one immediately above or below that particular response level than were participants with mild or moderate cognitive impairment. Approximately 67% of the time, participants in the no cognitive impairment group fixated upon the response option selected or the one immediately above or below, relative to 55% of the time for participants in the mild cognitive impairment group and 57% of the time for participants in the moderate cognitive impairment group. Participants with greater levels of cognitive impairment were more likely to fixate on none of the AOIs (categorised as “NOTHING”) prior to selecting their response. i.e. participants with no cognitive impairment focused on none of the AOIs approximately 2% of the time, compared with 18% of the time for those with mild cognitive impairment, and 23% of the time for those with moderate cognitive impairment.

Supplementary Information Table 1 presents the number and proportion of participants who exhibited specified patterns of eye movement during the gaze plot videos for each of the dimensions, for the entire sample as well as for each of the cognitive impairment subgroups. It can be seen that the largest proportion of participants (65.9%) began reading from the top of the mobility dimension, i.e. reading the heading for the mobility attribute. A lower proportion of participants showed this pattern for the dimensions presented later (i.e. Pain and Discomfort, of which only 51%, and Anxiety and Depression, where only 41% of participants began reading from the top of the question. There is also evidence of differences in the proportion of participants exhibiting this pattern within each cognitive impairment subgroup. For the Mobility dimension as an example, a higher proportion of participants without cognitive impairment (89%) began reading from the top of the question, compared to those with mild (65%), or moderate (50%) cognitive impairment. A lower proportion of participants moved sequentially through the response levels in order, moved backwards to a previous response level, or moved back and forth between many response. There was not a clear pattern in variation in proportions across the cognitive impairment subgroups. A much lower proportion of participants read only the top two response items (between 2.4 and 12.2% for the dimensions) and only a small proportion only read the response options further down the list (between 0 and 2.4%).

Supplementary Figures 4, 5 and 6 presenting the proportion of participants who did not record a fixation within the various AOI for the cognitive impairment subgroups. The exact proportions are provided in Supplementary Information Table 2. Some trends can be seen, where a higher proportion of participants did not record a fixation within the AOI for the Descriptors or Dimension Levels at the bottom of the screen (for example Severely or Unable or Extreme Problems) as compared to those at the top of the screen (for example, the No Problems level for each of the dimensions). However this did not reach statistical significance.

**Supplementary Table 1:** Frequency of use of reading pattern across dimensions for the entire sample and split according to cognitive impairment level

|  |  | **Number (%) exhibiting reading pattern** | | | | | | | |
| --- | --- | --- | --- | --- | --- | --- | --- | --- | --- |
| *Dimension* | *Cognitive impairment subsample* | **Begins reading top of question** | **Moves sequentially through the response levels** | **Moves backwards to a previous response level** | **No obvious logical pattern** | **Moves back and forth between many response levels** | **Revisits domain descriptor** | **Reads only the top two response options** | **Only reads lower down response options** |
| *Mobility* | Total | 27 (65.9) | 22 (53.7) | 18 (43.9) | 4 (9.8) | 8 (19.5) | 10 (24.4) | 2 (4.9) | 0 (-) |
|  | *None* | 8 (88.9) | 5 (55.6) | 6 (66.7) | 1 (11.1) | 2 (22.2) | 3 (33.3) | 0 (-) | 0 (-) |
|  | *Mild* | 13 (65.0) | 12 (60.0) | 8 (40.0) | 3 (15.0) | 2 (10.0) | 4 (20.0) | 1 (5.0) | 0 (-) |
|  | *Moderate* | 6 (50.0) | 5 (41.7) | 4 (33.3) | 0 (-) | 4 (33.3) | 3 (25.0) | 1 (8.3) | 0 (-) |
| *Self-Care* | Total | 19 (46.3) | 21 (51.2) | 8 (19.5) | 4 (9.8) | 7 (17.1) | 8 (19.5) | 1 (2.4) | 1 (2.4) |
|  | *None* | 8 (88.9) | 8 (88.9) | 3 (33.3) | 0 (-) | 2 (22.2) | 0 (-) | 0 (-) | 1 (11.1) |
|  | *Mild* | 8 (40.0) | 8 (40.0) | 2 (10.0) | 3 (15.0) | 1 (5.0) | 5 (25.5) | 1 (5.0) | 0 (-) |
|  | *Moderate* | 3 (25.0) | 5 (41.7) | 3 (25.0) | 1 (8.3) | 8 (66.7) | 3 (25.0) | 0 (-) | 0 (-) |
| *Usual Activities* | Total | 22 (53.7) | 13 (31.7) | 11 (26.8) | 6 (14.6) | 12 (29.3) | 13 (31.7) | 5 (12.2) | 2 (4.9) |
|  | *None* | 8 (88.9) | 4 (44.4) | 2 (22.2) | 0 (-) | 3 (33.3) | 4 (44.4) | 3 (33.0) | 1 (11.1) |
|  | *Mild* | 10 (50.0) | 6 (30.0) | 6 (30.0) | 3 (15.0) | 4 (20.0) | 5 (25.0) | 2 (10.0) | 1 (5.0) |
|  | *Moderate* | 4 (33.3) | 3 (25.0) | 3 (25.0) | 3 (25.0) | 5 (41.7) | 4 (33.3) | 0 (-) | 0 (-) |
| *Pain/Discomfort* | Total | 21 (51.2) | 16 (39.0) | 11 (26.8) | 7 (17.1) | 10 (24.4) | 7 (17.1) | 4 (9.8) | 0 (-) |
|  | *None* | 5 (55.5) | 3 (33.3) | 1 (11.1) | 0 (-) | 4 (44.4) | 3 (33.3) | 3 (33.3) | 0 (-) |
|  | *Mild* | 10 (50.0) | 8 (40.0) | 5 (25.0) | 4 (20.0) | 3 (15.0) | 1 (5.0) | 1 (5.0) | 0 (-) |
|  | *Moderate* | 6 (50.0) | 5 (41.7) | 5 (41.7) | 3 (25.0) | 3 (25.0) | 3 (25.0) | 0 (-) | 0 (-) |
| *Anxiety/*  *Depression* | Total | 17 (41.5) | 16 (39.0) | 10 (24.4) | 6 (14.6) | 14 (34.1) | 15 (36.6) | 3 (7.3) | 1 (2.4) |
|  | *None* | 6 (66.7) | 5 (55.6) | 3 (33.3) | 0 (-) | 3 (33.3) | 4 (44.4) | 2 (22.2) | 0 (-) |
|  | *Mild* | 7 (35.0) | 7 (35.0) | 3 (15.0) | 3 (15.0) | 7 (35.0) | 5 (25.0) | 1 (5.0) | 1 (5.0) |
|  | *Moderate* | 4 (33.3) | 4 (33.3) | 4 (33.3) | 3 (25.0) | 4 (33.3) | 6 (50.0) | 0 (-) | 0 (-) |


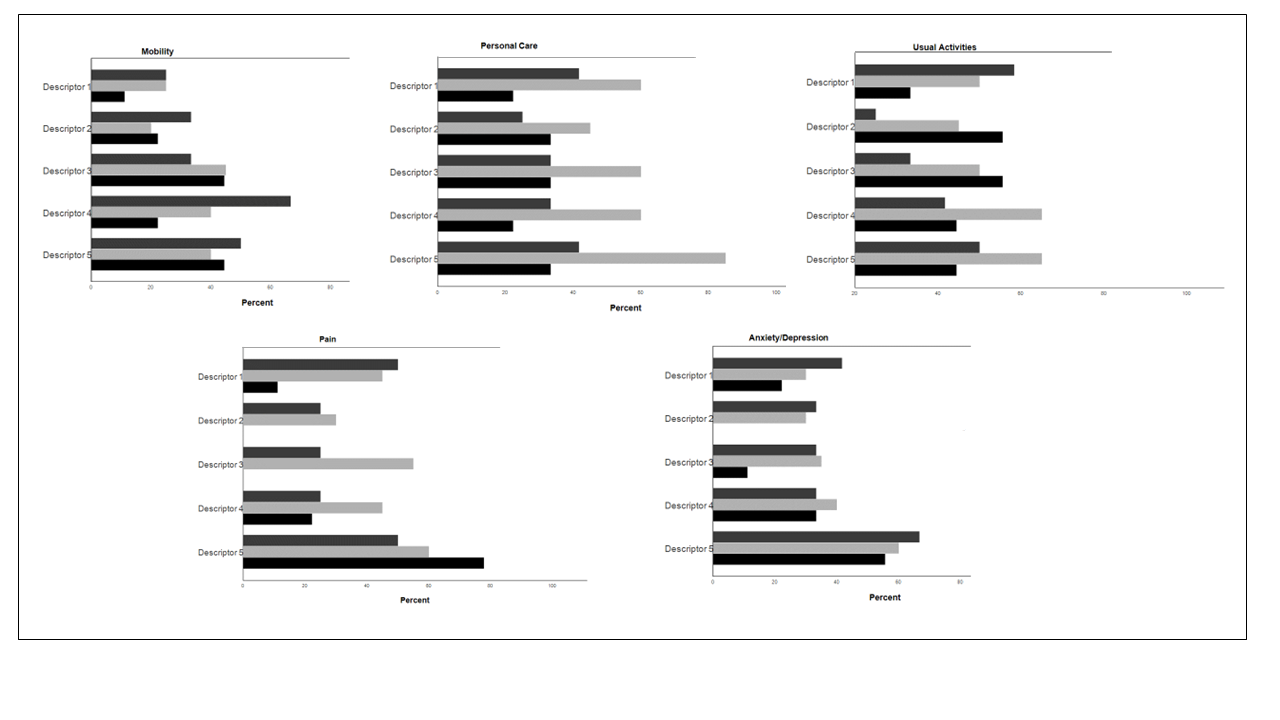
**Supplementary Figure 4:** Proportion of participants who who did not record a fixation within the Dimension Descriptor AOI for the cognitive impairment subgroups. Figure Legend: Dark Grey Bar indicates the no cognitive impairment subgroup, light grey indicates the mild cognitive impairment subgroup, and black indicates the moderate cognitive impairment subgroup.


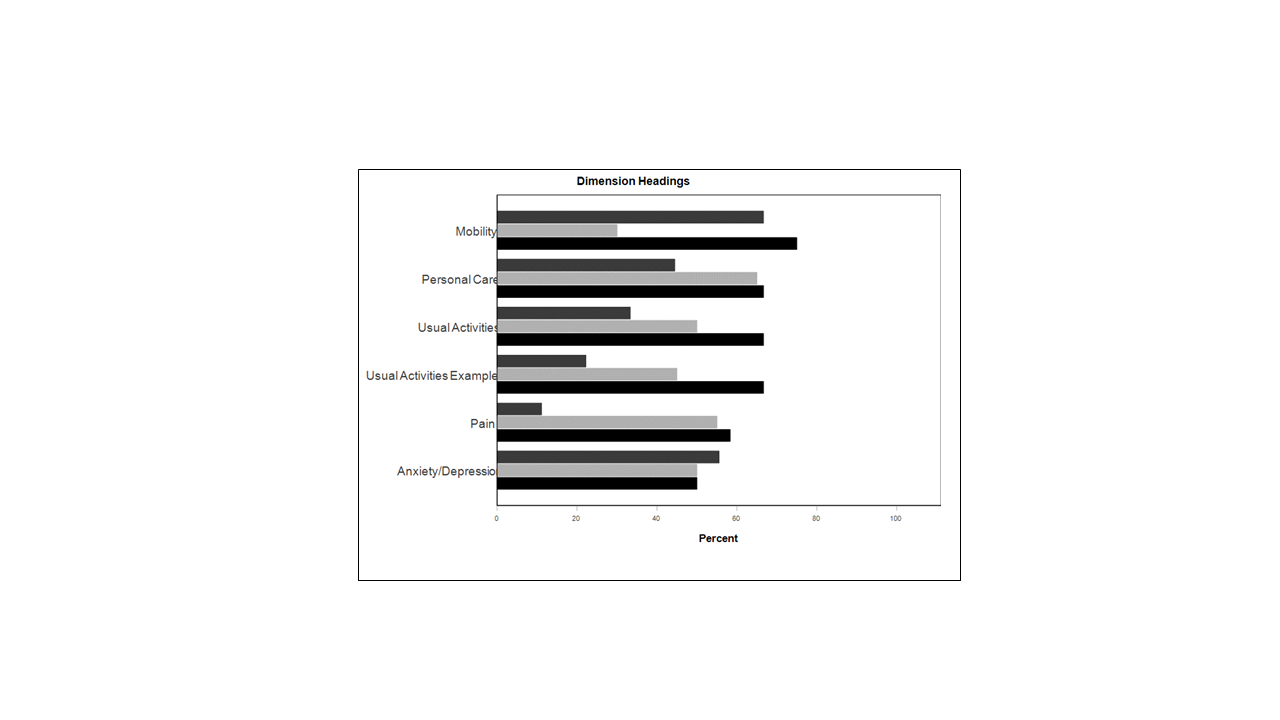


**Supplementary Figure 5**: Proportion of participants who who did not record a fixation within the Dimension Headings AOI for the cognitive impairment subgroups

Figure Legend: Dark Grey Bar indicates the no cognitive impairment subgroup, light grey indicates the mild cognitive impairment subgroup, and black indicates the moderate cognitive impairment subgroup.


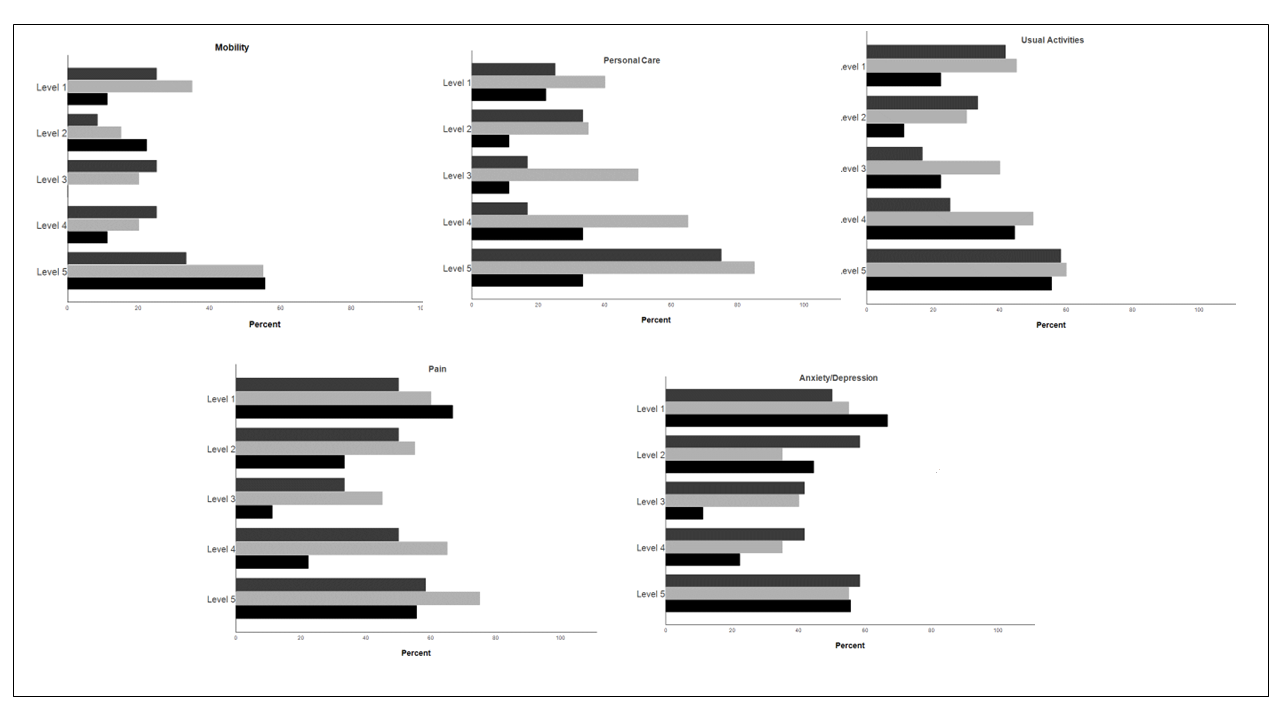


**Figure 6**: Proportion of participants who who did not record a fixation within the Dimension Level AOI for the cognitive impairment subgroups

Figure Legend: Dark Grey Bar indicates the no cognitive impairment subgroup, light grey indicates the mild cognitive impairment subgroup, and black indicates the moderate cognitive impairment subgroup.

**Supplementary Table 2:** Participants who did not record a fixation within the AOI, for the entire sample and cognitive impairment subgroups

|  | | **Number (%)** | | | | | | | | | | | | | |
| --- | --- | --- | --- | --- | --- | --- | --- | --- | --- | --- | --- | --- | --- | --- | --- |
| *Dimension* | *Cognitive impairment subsample* | **Dimension label** | **Example** | **Descriptors** | | | | | **Dimension Levels (e.g. No problems)** | | | | | **Bottom two Levels** | **Top Two Levels** |
|  |  |  |  | **1** | **2** | **3** | **4** | **5** | **No Problems** | **Slight** | **Moderately** | **Severely** | **Unable/ Extreme** |  |  |
| *Mobility* | Total | 21 (51.2) |  | 9 (22.0) | 10 (24.4) | 17 (41.5) | 18 (43.9) | 18 (43.9) | 11 (26.8) | 6 (14.6) | 7 (17.1) | 8 (19.5) | 20 (48.8) | 5 (12.2) | 6 (14.6) |
|  | *None* | 6 (66.7) |  | 1 (11.1) | 2 (22.0) | 4 (44.4) | 2 (22.2) | 4 (44.4) | 1 (11.1) | 2 (22.2) | 1 (11.1) | 1 (11.1) | 5 (55.6) | 0 (0) | 1 (11.1) |
|  | *Mild* | 6 (30.0) |  | 5 (25.0) | 4 (20.0) | 9 (45.0) | 8 (40.0) | 8 (40.0) | 7 (35.0) | 3 (15.0) | 4 (20.0) | 4 (20.0) | 11 (55.0) | 2 (10.0) | 3 (15.0) |
|  | *Moderate* | 9 (75.0) |  | 3 (25.0) | 4 (33.0) | 4 (33.3) | 8 (66.7) | 6 (50.0) | 3 (25.0) | 1 (8.3) | 3 (25.0) | 3 (25.0) | 4 (33.3) | 3 (25.0) | 2 (16.7) |
| *Self-Care* | Total | 25 (61.0) |  | 19 (46.3) | 15 (36.6) | 19 (46.3) | 18 (43.9) | 25 (61.0) | 13 (31.7) | 12 (29.3) | 13 (31.7) | 18 (43.9) | 29 (70.7) | 12 (29.3) | 1 (2.4) |
|  | *None* | 4 (44.4) |  | 2 (22.2) | 3 (33.3) | 3 (33.3) | 2 (22.2) | 3 (33.3) | 2 (22.2) | 1 (11.1) | 1 (11.1) | 3 (33.3) | 3 (33.3) | 1 (11.1) | 0 (0) |
|  | *Mild* | 13 (65.0) |  | 12 (60.0) | 9 (45.0) | 12 (60.0) | 12 (60.0) | 17 (85.0) * | 8 (40.0) | 7 (35.0) | 10 (50.0) | 13 * (65.0) | 17 ** (85.0) | 10 (50.0) | 1 (5.0) |
|  | *Moderate* | 8 (66.7) |  | 5 (41.7) | 3 (25.0) | 4 (33.3) | 4 (33.3) | 5 (41.7) | 3 (25.0) | 4 (33.3) | 2 (16.7) | 2 (16.7) | 9 (75.0) | 1 (8.3) | 1 (8.3) |
| *Usual Activities* | Total | 21 (51.2) | 19 (46.3) | 20 (48.8) | 17 (41.5) | 19 (46.3) | 22 (53.7) | 23 (56.1) | 16 (39.0) | 11 (26.8) | 12 (29.3) | 17 (41.5) | 24 (58.5) | 7 (17.1) | 2 (4.9) |
|  | *None* | 3 (33.3) | 2 (22.2) | 3 (33.3) | 5 (55.6) | 5 (55.6) | 4 (44.4) | 4 (44.4) | 2 (22.2) | 1 (11.1) | 2 (22.2) | 4 (44.4) | 5 (55.6) | 1 (11.1) | 0 (0) |
|  | *Mild* | 10 (50.0) | 9 (45.0) | 10 (50.0) | 9 (45.0) | 10 (50.0) | 13 (65.0) | 13 (65.0) | 9 (45.0) | 6 (30.0) | 8 (40.0) | 10 (50.0) | 12 (60.0) | 4 (20.0) | 1 (5.0) |
|  | *Moderate* | 8 (66.7) | 8 (66.7) | 7 (58.3) | 3 (45.0) | 4 (33.3) | 5 (41.7) | 6 (50.0) | 5 (41.7) | 4 (33.3) | 2 (16.7) | 3 (25.0) | 7 (58.3) | 2 (16.7) | 1 (8.3) |
| *Pain/ Discomfort* | Total | 19 (46.3) |  | 16 (39.0) | 9 (22.0) | 14 (34.1) | 14 (34.1) | 25 (61.0) | 24 (58.5) | 20 (48.8) | 14 (34.1) | 21 (51.2) | 27 (65.9) | 8 (19.5) | 2 (4.9) |
|  | *None* | 1 (11.1) |  | 1 (11.1) | 2 (22.2) | 1 (11.1) | 2 (22.2) | 7 (77.8) | 6 (66.7) | 3 (33.3) | 1 (11.1) | 2 (22.20 | 5 (55.6) | 0 (0) | 0 (0) |
|  | *Mild* | 11 (55.5) * |  | 9 (45.0) | 6 (30.0) | 11 (55.0) ** | 9 (45.0) | 12 (60.0) | 12 (60.0) | 11 (55.0) | 9 (45.0) | 13 (65.0) | 15 (75.0) | 6 (30.0) | 1 (5.0) |
|  | *Moderate* | 7 (58.3) |  | 6 (50.0) | 3 (25.0) | 3 (25.0) | 3 (25.0) | 6 (50.0) | 6 (50.0) | 6 (50.0) | 4 (33.3) | 6 (50.0) | 7 (58.3) | 2 (16.7) | 1 (8.3) |
| *Anxiety/ Depression* | Total | 21 (51.2) |  | 13 (31.7) | 10 (24.4) | 12 (29.3) | 15 (36.6) | 25 (61.0) | 23 (56.1) | 18 (43.9) | 14 (34.1) | 14 (34.1) | 23 (56.1) | 8 (19.5) | 2 (4.9) |
|  | *None* | 5 (55.6) |  | 2 (22.2) | 2 (22.2) | 1 (11.1) | 3 (33.3) | 5 (55.6) | 6 (66.7) | 4 (44.4) | 1 (11.1) | 2 (22.2) | 5 (55.6) | 0 (0) | 0 (0) |
|  | *Mild* | 10 (50.0) |  | 6 (30.0) | 6 (30.0) | 7 (35.0) | 8 (40.0) | 12 (60.0) | 11 (55.0) | 7 (35.0) | 8 (40.0) | 7 (35.0) | 11 (55.0) | 6 (30) | 1 (5.0) |
|  | *Moderate* | 6 (50.0) |  | 5 (41.7) | 4 (33.3) | 4 (33.3) | 4 (33.3) | 8 (66.7) | 6 (50.0) | 7 (58.3) | 5 (41.7) | 5 (41.7) | 7 (58.3) | 2 (16.7) | 1 (8.3) |
| *Note: * P ≤ 0.05, ** P ≤ 0.01, *** P ≤ 0.001. Tukey’s HSD and Dunnett’s method were used for making pairwise comparisons. Red stars refer to comparisons with `no cognitive impairment’ as a control, and green stars refer to `mild vs moderate’ comparisons.* | | | | | | | | | | | | | | | |
